# Volatile Metabolites Emission by In Vivo Microalgae—An Overlooked Opportunity?

**DOI:** 10.3390/metabo7030039

**Published:** 2017-07-31

**Authors:** Komandoor E. Achyuthan, Jason C. Harper, Ronald P. Manginell, Matthew W. Moorman

**Affiliations:** 1 Nano and Microsensors Department, Sandia National Laboratories, Albuquerque, NM 87185, USA; rpmangi@sandia.gov (R.P.M.); mmoorma@sandia.gov (M.W.M.); 2 Bioenergy and Defense Technology Department, Sandia National Laboratories, Albuquerque, NM 87185, USA; jcharpe@sandia.gov

**Keywords:** volatile organic compound, VOC, microalgae, in vivo emission, volatilome, volatilomics, volatome, volatile metabolites

## Abstract

Fragrances and malodors are ubiquitous in the environment, arising from natural and artificial processes, by the generation of volatile organic compounds (VOCs). Although VOCs constitute only a fraction of the metabolites produced by an organism, the detection of VOCs has a broad range of civilian, industrial, military, medical, and national security applications. The VOC metabolic profile of an organism has been referred to as its ‘volatilome’ (or ‘volatome’) and the study of volatilome/volatome is characterized as ‘volatilomics’, a relatively new category in the ‘omics’ arena. There is considerable literature on VOCs extracted destructively from microalgae for applications such as food, natural products chemistry, and biofuels. VOC emissions from living (in vivo) microalgae too are being increasingly appreciated as potential real-time indicators of the organism’s state of health (SoH) along with their contributions to the environment and ecology. This review summarizes VOC emissions from in vivo microalgae; tools and techniques for the collection, storage, transport, detection, and pattern analysis of VOC emissions; linking certain VOCs to biosynthetic/metabolic pathways; and the role of VOCs in microalgae growth, infochemical activities, predator-prey interactions, and general SoH.

## 1. Scope and Limitations

This review is focused on volatile organic compound (VOC) emissions from microalgae that were either living (in vivo), undergoing senescence or apoptosis, or perishing under predator attack. It is hypothesized that such emissions are relevant to the real-time detection of microalgae’s state of health (SoH). Consequently, this review excluded publications describing the collection of VOCs after lysis of the microalgae during extraction and analysis. Although the latter publications are valuable, the aim of this review was limited insofar as possible, to microalgal VOCs emitted under in vivo or otherwise natural living/dying conditions. To illustrate, the eukaryotic, unicellular green benthic microalga, *Ulothrix fimbriata*, was disintegrated using a freeze thaw cycle, followed by extraction of VOCs for analysis [1]. The prokaryotic cyanobacteria, *Nostoc* sp. was heat treated in methanol-water mixture followed by extraction of the volatiles using organic solvents; techniques which effectively destroyed the blue-green algae [2]. Destructive steam distillation followed by diethyl ether extraction was used to collect VOCs from the eukaryotic green alga, *Capsosiphon fulvescens* [3]. Such publications are considered here further only if there are aspects that are relevant to in vivo VOCs. For example, although VOCs were extracted after disintegrating *U. fimbriata* cells, such VOCs might be released by naturally dying cells, attracting predators [1] and exhibiting semiochemical behavior. Alternatively, microalgae might release specific VOCs under predator attack that are chemical cues of predation (disussed in detail under Section 5.5). In keeping with the title and scope of the review, emphasis was on VOCs, although microalgae do emit carbon-containing (CO, CO_2_) and non-carbon types of inorganic gases (example, H_2_, O_2_, N_2_O) that reflect upon the organism’s metabolic status. These gases are briefly discussed. This is not an exhaustive review, despite the citing of over 250 references. It was not possible to cite every paper published on VOC emanations from microalgae in vivo. Additional information is in the publications cited in this review.

## 2. Introduction

Detection of VOCs has a broad range of applications in civilian (for example, food science, cosmetics/fragrances, pharmaceutical, and environmental applications), military, medical, and national security arenas, resulting in the publication of several books on these topics [4,5,6,7]. Two well-known VOC applications are the breathalyzer test for intoxication and the use of police dogs for the detection of drugs, explosives, and crime scene evidence collection. The success of K9 (homophone for canine) units is due to the high number of odor receptor cells (ORCs) in the olfactory epithelium of dogs (~300 million in bloodhounds [8] compared to a human’s ~10 million). The rapid growth in VOC analysis is illustrated by several reviews within the last few years in *Metabolites* [9,10,11]. The explosive research output has spawned the new fields of volatilome (volatome) [12,13,14] and volatolomics (volatilomics) [15,16,17].

The diverse roles of microalgal VOCs in biochemistry, metabolism and physiology, ecology and environment, and modulating predator-prey interactions, as well as how such VOCs could be used to predict microalgae pond crashes due to predator attack, are amongst the key topics covered in this review. There is a great deal of interest in VOC emissions from microalgae due to their commercial value as food/feed, biofuels, and high value byproducts [18,19,20,21,22,23]. While considerable research has been carried out on VOCs extracted from microalgae using destructive techniques [1,2,3], this review focuses upon VOCs from living, growing, or naturally dying microalgae (in vivo VOCs). It is proposed that such VOC emissions might be useful for monitoring in real-time, the SoH of the microalgae. Where known, metabolic pathways leading to the emission of specific VOC molecules are described and contextualized regarding their biological/biochemical role(s) in the microorganism. This review may spark further interest in VOC profiles of microalgae in vivo for evaluating the role of volatilome/volatome in microalgae physiology, pathology, and ecology.

It is helpful to begin by presenting definitions of the two key topics in this review, namely, VOC and microalgae. In both cases, there is considerable scope for flexibility and ambiguity, depending on the defining agency. The term VOC includes a broad range of classes of small molecular mass (generally, ~200 g/mol) carbon chain or carbon ring-containing compounds, the smallest of which is methane, CH_4_. These compounds have high vapor pressure under ambient temperature (ambient conditions are defined as 20 °C and 101.3 kPa by the National Institute of Standards and Technology, NIST, USA). Due to their low boiling point, such compounds can readily transform from liquid phase to gaseous phase or from solid phase to gas phase (sublimation). VOCs can travel far from their source and pass through atmosphere, soil, and water. Due to their low-to-moderate hydrophilicity, VOCs can dissolve in water and disperse at the air–water interphase, exerting their infochemical effects widely, temporally and spatially. In terms of biologically-generated VOCs (biogenic VOC, BVOC), such volatiles represent secondary metabolites of the microalgae. The distinction between primary and secondary metabolites is that the former being constitutive, are continuously produced and are essential for maintaining life; secondary metabolites on the other hand may be derived from primary metabolites and are not directly involved in the growth, development, and reproduction of the organism. Here, the term VOC excludes gases such as carbon monoxide (CO) and carbon dioxide (CO_2_), but includes compounds with perhaps up to 15 carbon atoms and a boiling point of up to 260 °C, amongst the VOCs. Several different definitions exist for VOCs depending upon the organization (World Health Organization, WHO; European Union, EU; Environmental Protection Agency, EPA, USA; American Society for Testing and Materials International, ASTM International) responsible for issuing such definitions [24]. The general properties of VOCs are summarized in Table 1.

Microalgae also have debatable definitions. It has even been asserted that the term algae itself may have no formal taxonomic meaning [25], since algal taxonomy is continuously and rapidly evolving based on genetic and microscopic data. Algae have been described as a polyphyletic (organisms without a common origin, but arising along multiple and independent evolutionary lines) group of oxygen-producing, photosynthetic organisms. This group includes both macroalgae and microalgae [26]. According to phycologists, two criteria must be met to classify an organism as an alga: (a) it must contain chlorophyll *a*, which is capable of utilizing sunlight to generate chemical energy; and (b) it must possess a thallus (vegetative tissue) that, unlike plants, is not differentiated into roots, stem, and leaves [27]. Cyanobacteria (blue-green algae) are frequently included amongst microalgae, even though they are prokaryotic (lacking membrane-bound organelles), Gram negative bacteria [20,25,27,28]. There is evidence that plastids in algae and higher plants evolved from cyanobacteria through an endosymbiotic event with a host. Cyanobacteria and their VOCs are therefore considered here but not VOCs from macroalgae, except as noted.

**Figure 1:**
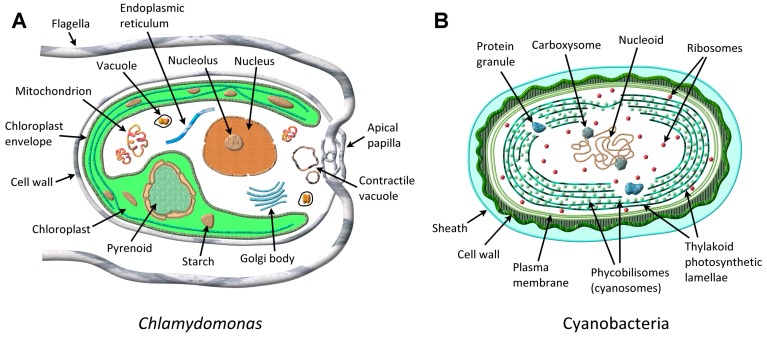
Schematic of the major components of eukaryotic (**A**) and prokaryotic (**B**) microalgal cells.

Microalgae, as the term denotes, are a diverse group of microscopic, typically unicellular, eukaryotic (consisting of membrane bound, cell function-controlling organelles such as nucleus, mitochondria, chloroplast, Golgi body, flagella, surrounded by plasma membrane, the *plasmalemma*, as shown in Figure 1A), plant-like photosynthetic microorganisms, capable of fixing atmospheric CO_2_ in the presence of sunlight and catalyzing its conversion into biomass for nutrition and growth [29]. Cyanobacteria (*Cyanophyceae*) are examples of prokaryotic microalgae (Figure 1B), whereas green algae (*Chlorophyta*) and diatoms for example, represent eukaryotic microalgae. Microalgae are present in both freshwater (lakes, rivers) and salt water (oceans), as planktonic (suspended) or benthonic (attached to the bottom) marine systems. Microalgae can also be terrestrial (soil microalgae). Microalgae size ranges from a few micrometers (μm) to >100 μm. It has been estimated that several thousands of microalgal species exist distributed across different genera. Microalgae, phytoplankton, and picophytoplankton (<1 μm) [25], form the base of the food chain in the marine environment, where oceans cover ~70% of the earth’s surface. Autotrophic microalgae require only inorganic salts, CO_2_, and light for growth and development, whereas heterotrophic microalgae, which are non-photosynthetic, need external sources of organic compounds and nutrients for growth. Mixotrophic algae can survive either by photosynthesis or acquire nutrients externally, depending upon the environmental conditions. Microalgae generate about one-half to two-thirds of the atmospheric O_2_. Microalgae occur as green, red, brown, gold, yellow-green, blue-green, and as diatoms. There are several sources available for a list of microalgae cultures including the AlgaeBase and the microalgae culture collection from the US Department of Energy (DOE) Aquatic Species Program, which is cataloged in the Solar Energy Research Institute (SERI) database, Golden, CO, USA. A brief description is supplied of the microalgal species whenever it is first mentioned in this review.

## 3. In Vivo Microalgae VOCs

### 3.1. VOC Production Conditions

**Figure 2:**
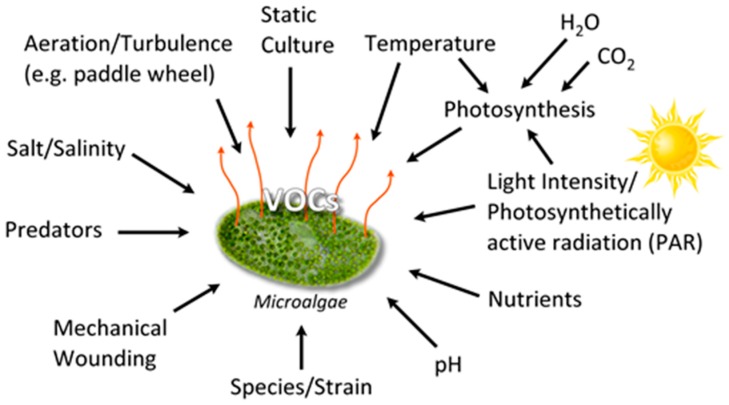
Factors influencing in vivo microalgae VOCs emission.

Algal VOCs (AVOC or biogenic VOC, BVOC), being secondary metabolites [30] are produced under various conditions (Figure 2). The VOC chemistry, production rate, and the quantity of emission(s) depend upon several biotic and abiotic factors summarized in Figure 2, such as growth phase, species/strain type, stress (seasonal changes, temperature, light intensity, pH, salinity), water, nutrients, gases (H_2_O, CO_2_, O_3_), aeration (mixing/turbulence) or static culture, and the presence of predators [31].

**Figure 3:**
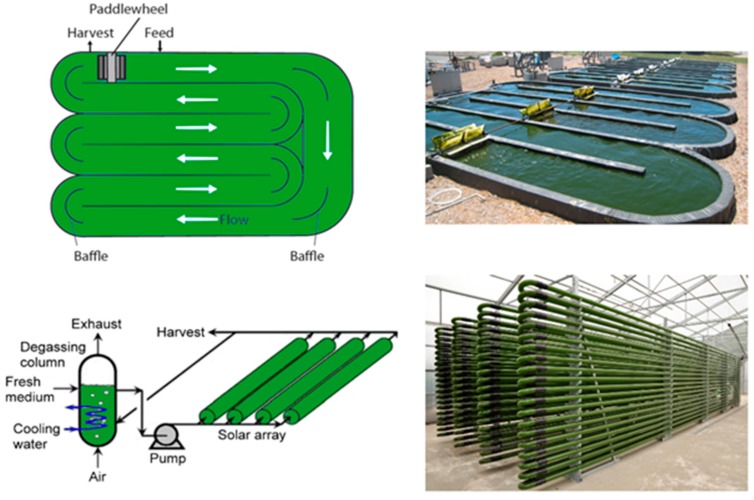
Schematic (**left**) and photographs (**right**) of ORP (**top panels**) and PBR (**bottom panels**). Adapted, with permission, from Biotechnology Advances 2007, 25, 294–306.

It is relatively straightforward to collect and study VOCs after lysing the microalgae using cell disruption techniques such as homogenization, sonication, freeze-thaw cycles, high temperature treatment, steam distillation, or extraction using organic solvents. On the other hand, extraction of VOCs from living (in vivo) microalgae is difficult and requires non-invasive techniques to avoid cell lysis. Furthermore, collection of emitted VOCs is more challenging depending on the culture, environmental, and climate conditions. Microalgae are generally cultured in the laboratory using flasks, and commercially cultivated in low-cost open raceway ponds (ORPs) or in closed, high-cost photobioreactors (PBRs) (Figure 3). While growth parameters can be carefully controlled and managed under laboratory culture conditions, this is less likely during microalgal commercial cultivation, and perhaps nearly impossible to manage in the microalgae’s natural marine habitat where conditions exist that are beyond human control. For example, phytoplanktons may be exposed to significant temperature changes due to vertical mixing in the water column of the marine environment which can change the organism’s metabolism [31]. Additionally, the low-cost advantage of ORPs is offset by the high risk of contamination and significant difficulties of maintaining both species control and contamination control. To date, microalgal cultures have failed to yield sustainable industrial-scale volumes of biomass at low-cost. Biological contamination is an important factor behind this failure [32].

Axenic cultures are important when assigning VOCs to a specific microalgae and not to a biological pollutant such as contaminating bacteria, fungi, rotifers, amoebae, or viral infections [33,34]. The VOCs may be produced by the invading organism or as a consequence of pathogen interactions with the host. Algae and bacteria have coexisted in the evolutionary timeline, and the two microorganisms affect each other’s physiology and influence the ecosystem where they dwell [28]. Although both microalgae and bacteria emit odorous VOCs, this review is focused mainly on VOC emissions from microalgae (and cyanobacteria). Regardless of whether the algae are grown in ORP or PBR, bacteria will inevitably and over time become a pollutant, which could cause sudden death of microalgal culture (pond crash) [35]. The VOC sampling regimen is also critical since the abundance of a particular microalgal species may not track directly with its AVOC levels or dynamics (asynchronous emission) [36]. Additionally, certain species that occur together in the natural habitat can produce similar VOCs, confounding assignment to a specific microalga. Several AVOCs also exist as more than one isomer, whereas biological activity might be confined to just one due to such considerations as conformational flexibility, stability, stereochemistry, or enantiomer blend [36]. The biosynthesis of AVOCs can vary within and between taxa. Such variations are regulated by endogenous and exogenous processes. Despite these challenges, AVOC metabolomics/volatilomics is important to microalgae’s biology, with commercial, societal, and global implications.

### 3.2. Inorganic Volatiles

Oxygen (O_2_) production is the most important step in any aerobic bioprocess, and this is true of microalgal respiration as well [37]. Photosynthetic microalgae produce approximately one-half to two-thirds of the molecular O_2_ in the atmosphere, and life on Earth evolved from, and now depends upon, such O_2_ generation. Equally important, microalgae consume CO_2_ (CO_2_ fixation), an important greenhouse gas (GHG) from the atmosphere. Molecular hydrogen (H_2_) can be produced by direct and indirect biophotolysis, which is suitable for solar energy to H_2_ transformation [37].

Nitrous oxide (N_2_O) is a potent GHG, a powerful scavenger of stratospheric ozone (O_3_) [38], and its emission from microalgae was summarized previously [39]. N_2_O was produced by several axenic cultures of microalgae, *Chlorella vulgaris*, *Chlorella rubescens*, *Chlorella homosphaericas*, *Scenedesmus obliquus*, *Coelastrum* spp., and *Chlorococcum vacuolatum*, along with three different cyanobacteria (*Nostoc* spp., *Aphanocapsa* PCC6308, and *Aphanocapsa* PCC6714). The microalgal cultures had been grown at 25 °C, under continuous irradiance (8–11.3 W/m^2^) for 18 h. All cultures had been checked for bacterial contamination and only data from bacteria-negative cultures were considered for analysis. Headspace gas was analyzed by gas chromatography (GC) coupled to thermal conductivity detector (TCD) and mass spectrometer (MS). It was suggested that microalgae play a role in aquatic N_2_O systems and probably in the overall global N_2_ balance [40,41].

Florez-Leiva et al. [42] measured N_2_O from *Nannochloris* (Chlorophyceae, green microalgae) monocultures grown in ORP with natural illumination and constant aeration using an airlift recirculation system. Water temperature, pH, and salinity were recorded, and gas emission was monitored over 46 days on a continuous basis. N_2_O was quantified using a GC-electron capture detector (ECD) using helium headspace equilibration. Among the emitted gases, N_2_O was emitted in the highest amount at a rate of 8 to 600 μmol/m^3^. N_2_O production abruptly peaked during senescence. A positive correlation between N_2_O production and chlorophyll *a* content suggested that a phototrophic pathway might trigger N_2_O generation, presumably by oxic NH_4_ oxidation.

Fagerstone et al. [43,44] reported N_2_O production by *Nannochloropsis salina* (chlorophyll *a* containing green microalga, but without chlorophyll *b* or *c*) grown under laboratory conditions, but simulating both ORP and PBR, with diurnal light–dark cycling. Microalgae were grown with shaking (140 revolutions per minute, rpm) at 23 °C, with 16 h light (90 μmol/m^2^/s) and 8 h dark periods. Headspace samples were collected in 8 h increments over the course of four days. Gas emission was quantitated using Fourier transform infrared spectroscopy (FTIR). Gas emission was elevated during the dark cycle but emission was minimal during the light periods. Anoxic N_2_O production was attributed to denitrifying bacteria, whereas oxic N_2_O was assigned to the microalgae.

Axenic cultures of *Chlorella vulgaris* (eukaryotic, unicellular, green algae) cultivated under laboratory conditions and in ORP were both shown to be capable of N_2_O production [45,46]. Microalgal cultivation involved light (82 W photosynthetically active radiation (PAR)/m^2^ for lab conditions; 92 μE/m^2^/s for ORP) and dark cycles at 25 °C. Headspace samples were analyzed by GC-ECD. Gas production was rapid initially during the first 4 h, and thereafter there was a linear N_2_O emission period that lasted for more than 24 h. The N_2_O production was dependent on nitrate reductase activity of *Chlorella vulgaris*, presumably involving nitrite reduction into nitric oxide (NO) which then converted to N_2_O. Although the authors did not extrapolate these findings to N_2_O emissions in the marine ecosystems, they did point out the impact a potentially open system of microalgae mass cultivation might have upon global biogeochemical N_2_ cycle and the atmospheric N_2_O budget. This is especially noteworthy, since the global warming potential (GWP) of N_2_O is 300 fold higher, on a molecule-by-molecule basis, relative to CO_2_ on a 100-year timescale [47].

Selenium (Se) chemically resembles sulfur (S) in that both elements occupy the same column in the periodic table, group VIA. Both have six valence electrons in the *ns* and *np* orbitals in the same arrangement of their outermost energy levels. Biochemically, Se is an essential trace element that is incorporated into selenoproteins as selenocysteine. Se salts are toxic environmental pollutants. The biogeochemistry of Se-VOC and volatile organic sulfur compound (VOSC) are thus closely linked since both can be converted to reduced- and alkylated-volatiles. Due to their similar chemical properties, sulfur can interfere with the uptake/absorption of selenium by microalgae.

In one study, *Chlamydomonas reinhardtii* (single cell green microalga) cultures (1 × 10^6^ cells/mL) were transferred to gas-tight solid phase microextraction (SPME) vials and the vials were crimped with a polytetrafluoroethylene (PTFE) septum. The cells were exposed to Se or S source for three days. Then the cells were placed in a 23–25 °C incubator with shaking (90 rpm) under constant illumination (100–120 μE_in_/m^2^), and Se-VOC and VOSC were measured using direct immersion (DI)-SPME followed by GC-MS analysis. There were increasing amounts of both Se-VOC and VOSC volatiles being produced under these conditions with the liberation of up to 500 μg/L of dimethyl-selenide (DMSe). However, the microalga underwent senescence within the VOC sampling periods of 3–9 days, owing to an absence of gas exchange in the gas-tight SPME vials [48]. Such inorganic to inorganic–organic hybrid volatile conversion is not surprising since biomethylating activity has been demonstrated in both freshwater and marine phytoplanktons [49].

Axenic culture of *Chlorella* sp. growth medium was supplemented with 20 μM Se, a concentration that was relevant to wetlands, but one that was non-toxic to the microalgae. Following 24 h incubation in the presence of antibiotics to prevent bacterial contamination, the gas-washing bottle containing the culture was connected to a second bottle containing NaOH-H_2_O_2_ mixture to trap the volatiles emitting from the culture bottle. The setup was maintained at 22–24 °C in a sunlit glasshouse with 16 h light period. At timed intervals the trap was sampled for Se emissions using flame atomic absorption spectroscopy. *Chlorella* converted toxic Se into volatile DMSe at rates which were several orders of magnitude higher than other wetland macroalgae or even plants. It was concluded that this “hypervolatilization” of Se by *Chlorella* may represent a new detoxification response by the microalgae [50]. A similar detoxification mechanism was also observed with three different microalgal species, *Ankistrodesmus* sp., (freshwater, long needle/spindle shaped green microalgae); *Chlorella vulgaris*, and *Selenastrum* sp., (freshwater, green microalgae), all of which were capable of metabolizing Se to DMSe. The DMSe was present in the headspace of the microalgal cultures as detected by GC-flame ionization detector (FID) [51].

### 3.3. VOCs-Containing Sulfur

**Figure 4:**
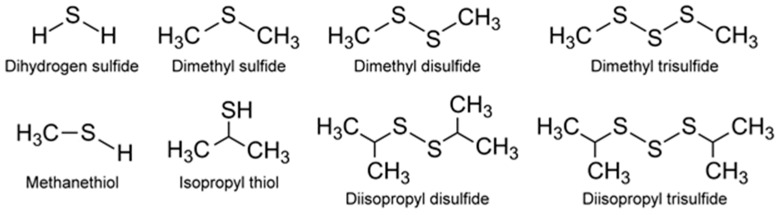
Chemical structures of microalgal key VOSCs.

Appropriately enough, VOSCs were perhaps the first odorous compounds to be systematically detected from algae. Paul Haas [52] observed in 1935 the malodor from decomposing seaweeds that was absent from natural, healthy seaweeds and correlated these malodors to VOCs emitted by the decaying *Polysiphonia fastigiata*, a small reddish-brown, filamentous macroalgae. He extracted the VOCs from the organism using a series of solvents (water, alcohol, acetone) and identified methyl sulfide being emitted during decomposition of the algae as at least one of the VOCs responsible for the malodor. More recently, a specialist group on taste and odor (T&O, T/O), belonging to the International Association on Water Pollution Research and Control (IAWPRC), listed the odorous algal culture collections worldwide in order to systematically study their biogenic origins. This list included microalgae (blue-green algae, cyanobacteria, green algae, *Chrysophyta*, *Chlorophyta* and diatoms), and macroalgae [53]. Since the pioneering work of Haas [52], there have been numerous studies of VOSCs (Figure 4), and some of these from in vivo microalgae are summarized below.

The VOSCs have important implications in the arena of global sulfur cycle [54] and climate [55], since algae are estimated to produce approximately 50% of the biogenic sulfur that is emitted into the atmosphere annually [56]. Additionally, alkane thiols such as methanethiol and isopropylthiol are intensely malodorous compounds that are known to be emitted by cyanobacteria. In fact, cyanobacterial mats are important sources of methanethiol and *Microcystis aeruginosa* (freshwater cyanobacteria) has been identified as a major emitter of isopropylthiol [55]. This microalga is a common toxic algal bloom-forming microorganism capable of causing considerable ecological and economic damage [57]. The emission of VOSCs might be a cue to early detection of microalgae, thus enabling preventive actions to be implemented.

**Figure 5:**
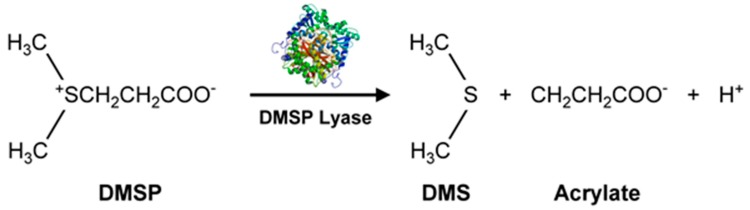
DMSP reaction liberating the volatile DMS product. A computer representation of the molecular level ribbon structure of DMSP lyase enzyme is shown above the reaction arrow.

Marine microalgae produce secondary organic aerosols (SOA), some of which involve VOSCs such as methane sulfonic acid (from dimethyl sulfide, DMS, H_3_C-S-CH_3_). The SOAs affect Earth’s climate by forming haze which blocks heat and impacts the marine boundary layer (MBL). Sulfates are important constituents of SOAs. Several microalgae like *Emiliania huxleyi* [58] (marine photosynthetic coccolithophore microalgae, largely found in subarctic ocean waters) and marine unicellular flagellate such as *Isochrysis galbana* [59], produce significantly more dimethylsulfoniopropionate (DMSP, precursor for DMS) than diatoms. Such microalgae could play an important role in marine sulfur cycling. Methanethiol and DMS are amongst the largest concentrations of BVOCs in the global sulfur cycle. A pathway for DMS production is by DMSP lyase acting on DMSP (Figure 5) [54,56].

The DMSP arises from the sulfur-containing amino acid methionine, initially from the enzymatic action of methionine decarboxylase and subsequently undergoes decarboxylation, oxidation, and methylation reactions to yield the final product [54]. Additionally, demethiolation of DMSP leads to methanethiol (H_3_C-SH) [54] which can be converted to DMS by methylation. The malodorous DMS is a low boiling point (~37 °C) liquid. Due to its high volatility, DMS can be detected using GC-flame photometric detector (FPD) or GC-MS. In addition to these biological and chemical roles, DMSP also functions as an osmolyte agent [60] by combating osmotic shocks to the microorganism arising from the varying salinities that may be encountered in the marine environment. Both DMS and DMSP also function as antioxidants [61], protecting the microalgae against damaging oxidizing radicals in the environment. DMS is released into the ocean waters in very high amounts of >10^7^ tons/year [62]. It can then be transported from the ocean surface into the atmosphere where it undergoes secondary, abiotic chemical reactions involving hydroxyl and nitrate radicals to generate a variety of degradation products such as CO_2_, carbonyl sulfide (COS), dimethyl sulfoxide (DMSO, (CH_3_)_2_SO), dimethyl sulfone (C_2_H_6_O_2_S), and organic acids of sulfur [54]. Furthermore, DMSP can undergo a non-enzymatic elimination reaction in the presence of base that also leads to the formation of DMS and acrylic acid. These enzymatic and non-enzymatic reaction products are capable of acting as grazing deterrents to the zooplankton, presumably due to DMS, but also attributed to the biocidal activity of acrylic acid [63,64]. Within the intact cell, the enzyme (DMSP lyase) and the substrate (DMSP) are segregated. Upon cell lysis due to grazing by a predator, the two come into contact, leading to the release of the concentrated acrylate which deters further grazing of *Emiliania huxleyi* by the protozoan, *Oxyrrhis marina* (dinoflagellate marine heterotroph) [65]. Thus, all these VOSCs play an important role in geochemical processes with global implications [63] well beyond their roles in the marine microalgae’s biology and biochemistry. This is supported by DMSP lyase in *Emiliania huxleyi* and invoking the possibility of using enzyme activity levels for the quantification of biogeochemical contributions of the microalgae and relating them to global DMS production [62]. Other sulfur compounds of potential climate impact include dimethyl disulfide (DMDS), dimethyl trisulfide (DMTS), isopropylthiol, diisopropyl disulfide, and diisopropyl trisulfide [55] (Figure 4).

In an early study of VOSCs from microalgal headspace, bacteria-free axenic cultures of several microalgae (*Gyrodinium cohnii*, eukaryotic dinoflagellate; *Amphidinium carterii*, dinoflagellate; *Cyclotella nana*, marine, freshwater diatom; *Glenodinium* sp., marine dinoflagellate; *Nannochloris oculata*, *Polytoma urella*, colorless, freeliving, flagellate chlorophyte; *Scenedesmus obliquus*, colonial, nonmotile green algae; *Chlorella vulgaris*; *Chlorella pyrenoidosa*; *Euglena gracilis*; and *Astasis longa*, eukaryotic uniflagellate) were all grown in chemically defined synthetic media. The headspace was sampled for volatiles during the growth phase. The sample was fractionated into an absorption train consisting of three parts for identifying specific VOSCs: thioether (using mercury (II) chloride, HgCl_2_); mercaptan (using mercury cyanide, Hg(CN)_2_); and hydrogen sulfide, H_2_S (using zinc acetate, Zn(O_2_CCH_3_)_2_) [66,67]. Despite early time period of this paper, an innovative system was employed for the identification of headspace volatiles from several microalgal cultures, since GC systems were still in an early phase of development in the US [68] when this work [66,67] was reported.

Blue-green algal mats collected from a hot spring in Yellowstone National Park (USA) were placed in vials containing spring water and incubated at 55 °C (mimicking in situ, hot spring temperature) under light and dark conditions. The algal mat underwent rapid decomposition, especially in the top 3 mm segment. Headspace VOCs were collected and analyzed using GC-FPD and contained only traces of DMS; instead copious amounts of methyl mercaptan and H_2_S were measured [69]. Furthermore, VOSC production was considerably lower under light conditions relative to dark incubations. These changes were attributed to the microalgae converting to aerobic metabolism due to O_2_ production [69]. As the blue-green algal mat grew upwards, the algae beneath entered the dark phase and began to decompose. Decomposition decreased (along with VOSC production) under aerobic, light incubation conditions. Because of growth and self-shading effects, photosynthesis occurred only in the top 3 mm of the mat. Beneath this zone, decomposition took place, accompanied by the emission of VOSCs. It is hard to draw additional conclusions due to a lack of identification of the microalgal species populating the mat and perhaps the xenic nature of the culture. In a larger study involving 123 phytoplankton clones from 12 different microalgal classes, the VOSC emissions were examined using headspace gas analysis by GC-FPD. Once again, since all cultures were xenic, the results have to be interpreted cautiously. It was noted that all species produced DMS. A strong correlation was observed between the taxonomic position of the phytoplankton and the production of DMS [59].

Oceanic DMS levels vary seasonally and regionally, dependent upon the population density of the microalgal species. For example, diatoms which produce relatively low levels of DMS, might yet be significant contributors to the ocean’s VOSCs budget due to the microalga’s population density. Release of DMS into the marine environment might depend on salinity, since in lab experiments, intracellular DMSP concentrations increased with an increase of salt concentration in the media [59]. Release of DMS from DMSP could arise from DMSP lyase activity of microalgae such as *Emiliania huxleyi* [59,70] or from co-existing bacterial action. As algae age, entering the senescent phase [70], or getting eaten by predators, intracellular stores of DMS and DMSP are released into the water column where DMS could be oxidized to sulfur dioxide (SO_2_), and then to sulfuric acid (H_2_SO_4_), resulting in acid precipitation. A portion of the DMS could get converted to sulfate aerosols which serve as cloud condensation nuclei (CCN), and are critical to cloud formation [59].

### 3.4. VOCs-Containing Halogens

**Figure 6:**
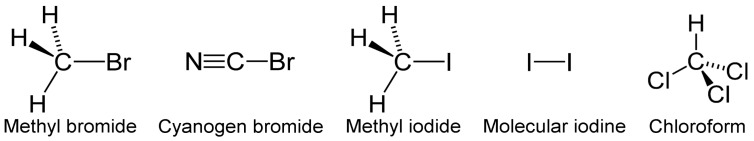
Chemical structures of key microalgal VHCs.

Volatile halocarbons (VHCs) (Figure 6) incorporate halogen atoms of the same or dissimilar type, linked by covalent bonds. In air, VHCs are implicated in the destruction of the UV-protective ozone layer. In water, they appear as trihalomethanes (THM) due to chlorination disinfection or from release of waste products, becoming environmental pollutants and suspected carcinogens. The VHCs released into marine waters by the microalgae may be transported into the atmosphere, where they directly or indirectly affect global climate. Deleterious effects of VHCs were confirmed by the O_3_ destroying effects of chlorofluorocarbons (CFCs) in the troposphere and stratosphere [71]. The CFCs are split by exposure to ultraviolet (UV) radiation, resulting in the release of chlorine atom, a long-lived radical which converts ozone into molecular oxygen:

CCl_3_F → CCl_2_F + Cl

Cl + O_3_ → ClO + O_2_

Other photolabile halogens emitted by microalgae are tribromomethane (CHBr_3_), dibromomethane (CH_2_Br_2_), iodomethane (CH_3_I), diiodomethane (CH_2_I_2_), iodoethane (C_2_H_5_I), bromoiodomethane (CH_2_BrI), chloroiodomethane (CH_2_ICl), iodine (I_2_) bromochloromethane (CH_2_BrCl), bromodichloromethane (CHBrCl_2_), chloroflorm (CHCl_3_), and dibromochloromethane (CHBr_2_Cl) [72,73]. Although radicals from these VHCs might be gases with a life span of several days to weeks at mid and high altitudes, in the tropics such VHCs could ride convection currents and be carried into the troposphere and stratosphere [74] and participate in ozone depletion events (ODEs) in the troposphere, acting as CCN for larger particles with global climate-changing impact [75]. For example, Br_2_ and I_2_ pulses were suggested as ODEs [76,77].

Reactive halogen species (RHS) are integral to biogenic emissions. It was estimated that marine algae contribute approximately 70% of the Earth’s bromoform [78]. Microalgae were proposed as the major contributors to global biogenic production of halocarbons due to their wide spread distribution and abundance in the marine environment, despite production rates being lower than macroalgae [79,80]. Microalgal production of VHCs is influenced by the usual factors such as C, N, nutrient availability, pH, illumination, photosynthetic activity, oxidative stress, mechanical wounding, predator grazing, salinity, and so on (Figure 2) [81]. Furthermore, the rates of VHC biosynthesis and exudation into the environment might depend on the type of microalgal species, its geographical location, and seasonal variations. For example, a decrease in seawater pH may lead to an increase in VHC emission [81]. In this context, the pH of ocean waters is reported to be decreasing at a rate of 0.0019 units per year, leading to a phenomenon known as ocean acidification (OA) [82,83]. Microalgae may also indirectly contribute to VHC production. For example, microalgae exude copious amounts of dissolved organic matter (DOM) into the marine environment [84,85] which plays a role in the ocean’s biogeochemistry. The DOM includes VHCs produced on the marine surface through a range of biological and photochemical processes, creating a strong flux. The DOM itself can react abiotically with light and ozone in a photochemical degradation reaction to further generate halocarbons. These halocarbons undergo water–air gas exchange, releasing VHCs into the atmosphere [84,85].

Despite wide ecological distribution and the capability to produce RHS, VHC emissions by microalgae “are still poorly known” [86] and not well understood [80]. “Waste microalgae”, defined as algae from laboratory algal growth systems, underwent I_2_ mobilization which was linearly correlated with carbon emission, suggesting the formation of organoiodine [87]. Arctic microalgae (predominantly pennate diatoms) were found to emit significant amounts of bromoform [76]. The global contribution of organic bromine compounds (5–80 × 10^9^ g/year) was suggested to be at levels that were comparable to macrophyte and anthropogenic sources [76]. Several classes of halomethanes were produced by different types of cyanobacteria and other microalgae such as diatoms, chlorophytes, and coccolithophorids [80]. Biosynthesis of mono-halomethanes is catalyzed by methyl transferase and occurs in *Pavlova pinguis*, a marine eukaryotic microorganism. Polyhalomethanes are also formed via catalysis by haloperoxidase [88]. During haloperoxidase reactions, one molecule of H_2_O is lost. Here, H_2_O_2_ oxidizes halide ion (X) yielding hypohalous acid, which then halogenates the electron-rich organic compounds to produce polyhalomethanes:

*S*-adenosyl-l-methionine (SAM) + X^−^ → (methyl transferase) → CH_3_X + SAM (*minus* methyl)

H_2_O_2_ + X^−^ + H^+^ → (haloperoxidase) → HOX + RH → R-X

Methyl iodide was produced by the phytoplankton *Nannochloropsis salina* (CCMP1776) during a reaction catalyzed by halide ion thiol methyl transferase (HTMT). Significant amounts of methyl iodide were produced in these reactions (668.1 pmol/g biomass/day) despite the fact that a fraction of the product was converted to methyl chloride and lost to sea water [89]. In a systematic study, axenic cultures of *Porphyridium purpurem* (mesophilic, unicellular red microalga) were grown with orbital mixing (50 rpm) under 17:7 h cycles of light (20 μmol quanta/m^2^/s) and dark at 22 °C. Headspace halocarbons were extracted and analyzed using GC-MS. Only chloroform and methyl iodide were produced in measurable amounts in these experiments. Chloroform production was maximal during the logarithmic growth phase of the microalgae [90].

Diatom cultures of *Mediopyxis helysia* and *Porosira glacialis* were incubated at 16 °C under a 12 h light (40 μmol PAR) and 12 h dark cycle inside a 10 L glass tube, with constant mixing under a continuous flow of synthetic air (3.4 L/min) over the microalgal suspension. The chamber’s outflow gas was sampled and the halocarbons were detected using GC-MS. The following VHCs were emitted from both diatom cultures: iodomethane, iodochloromethane, diiodomethane, iodite, iodate, iodine, and bromoform [77]. Axenic cultures of the phytoplanktons *Nitzschia* sp. (pennate marine diatom), *Navicula* sp. (primarily aquatic, eukaryotic, photosynthetic diatom), *Thalassiosira pseudonana*, (eukaryotic marine diatom), *Emiliania huxleyi*, and *Dunaliella tertiolecta* (unicellular, rod-to-ovoid shape green microalgae), all produced iodide. Iodide production was sensitive to environmental conditions, and interestingly enough, was also sensitive to strain level differences [91], which might be helpful for identifying microalgal strains using iodide as a volatile biomarker.

Some microalgae such as *Nitzschia* sp., *Navicula* sp., *Porosira glacialis* (unicellular diatom) and *Phaeocystis* sp. (wide spread, eukaryotic phytoplankton), were shown to produce methyl iodide in lab monocultures by headspace gas sampling [79]. It was unclear whether the cultures were axenic. Scarratt and Moore [92] examined cultures of several marine phytoplanktons: *Emiliania huxleyi*, *Prorocentrum* sp. (photosynthetic dinoflagellate), *Tetraselmis* sp. (eukaryotic, photosynthetic *Chlorophyta*), *Phaeocystis* sp., *Isochrysis* sp. (brown-golden marine microalga), *Porphyridium* sp. (red microalga, *Rhodophyta*), *Synechococcus* sp. (unicellular cyanobacterium), *Chaetoceros calcitrans* (fast growing diatom), *Thalassiosira weisflogii* (centric diatom, unicellular microalga), and *Phaeodactylum tricornutum* (diatom existing in different shapes, morphotypes). All cultures were axenic, with the exception of *Porphyridium* sp. and *Prorocentrum* sp. Cultures were bubbled with ultra-high purity (UHP) air and grown in 17 h light (150 μmol/m^2^/s) and 7 h dark cycles. The effluent air was cryotrapped and analyzed by GC-MS or GC-ECD [92]. All microalgae produced methyl bromide and methyl chloride. In addition, *Thalassiosira weisflogii* and *Phaeodactylum tricornutum* also produced C_2_H_5_I, CH_2_IBr, CH_2_ICl, CH_2_I_2_, and CH_3_I [92].

Finally, the production of cyanogen bromide (CNBr) in the headspace of the marine benthic diatom *Nitzschia cf pellucida*, provided a clue regarding the importance of halocarbons to microalgae [93]. CNBr was an allelochemical, a potent inhibitor of this diatom’s competitors in the environment. Exposure of the diatom’s competitors—*Cylindotheca closterium* (unialgal phytoplankton), *Navicula arenaria*, and *Entomoneis paludosa* (diatom)—to CNBr caused chloroplast bleaching, decreased photosynthetic efficiency, growth inhibition, and cell death after 24 h. It appeared that *Nitzschia cf pellucida* does a self-cleaning of its surroundings by producing CNBr, thereby clearing a space around itself for growth and development, as part of the organism’s daily housekeeping activities. In addition to CNBr, the head space of *Nitzschia cf pellucida* contained a diverse mixture of VHC metabolites [93] whose identities and biological functions remain to be elucidated.

### 3.5. Other VOCs

**Figure 7:**
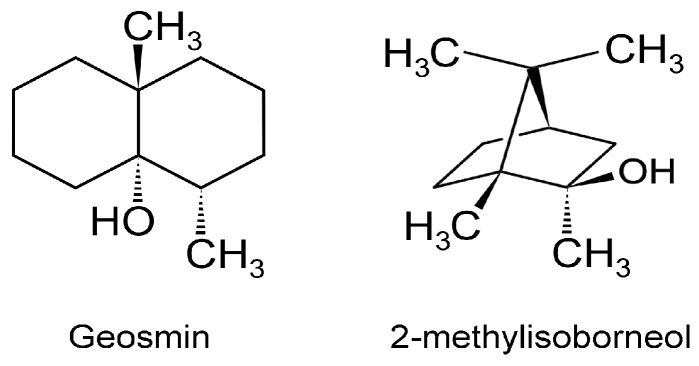
Chemical structures of two key T&O compounds, geosmin, and 2-MIB.

Marine VOCs, like terrestrial VOCs, are ubiquitous in the oceans, and these emissions are influenced by biological, photochemical, meteorological, and anthropogenic factors, leading to spatio-temporal variations [94]. Oxygenated VOCs (OVOCs) play a role in atmospheric processes. Methanol is an important OVOC with an important role in O_3_ formation in the troposphere [95]. Given that the Earth’s surface is covered by ~70% water, and the oceans hold about 97% of all Earth’s water, marine aerosols and their associated VOCs have received increasing attention [96]. Thus, it is important to not view VOCs as a threat to ecology, but to understand their multifaceted appearances and functions, biotic and abiotic. This will provide a better foundation for productively tasking VOCs for practical applications. Although several VOCs are odorous (VOSCs, VHCs), two are of concern since these appear in potable water [97]. The two secondary terpenoid compounds are geosmin ((4S, 4aS,8aR)-4,8a-dimethyl-1,2,3,4,5,6,7,8-octahydronaphthalen-4a-ol; C_12_H_22_O) and 2-methylisoborneol (2-MIB) ((1R-exo)-1,2,7,7-tetramethylbicyclo[2.2.1]heptan-2-ol; C_11_H_20_O) [98] (Figure 7).

**Figure 8:**
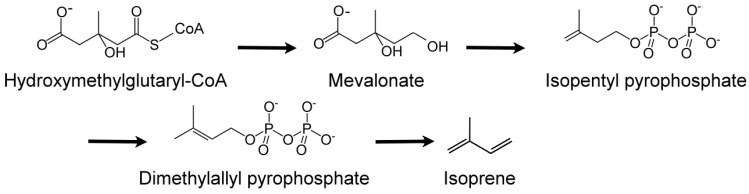
Mevalonate pathway for isoprene production.

Humans are sensitive to the presence of these two malodorous aquatic pollutants since the odor threshold concentration (OTC) for geosmin and 2-MIB are in the range of 4 to 10 ng/L. Thus, the human nose can be a ‘biosensor’ for the ‘earthy/muddy/moldy’ odor of these VOCs. Cyanobacteria are probably exclusive to microalgae generating these VOCs [36,97,98,99], either through cell leakage or cell lysis during mechanical wounding or predator grazing. These two stable VOCs resist conventional water treatment techniques and are capable of being transported through the environment in which they are produced as well as affecting water quality distant to their origin. Eutrophication exacerbates the situation by encouraging algal blooms, further impacting ecology and water quality. A 2007 microalgal infestation of Lake Taihu (China), led to a shutdown of water supply to over two million residents for five days [98], highlighting the importance of early detection and remediation of T&O VOCs such as geosmin and 2-MIB. In the cyanobacterium *Synechocystis* sp., geosmin was shown to be biosynthesized by the isoprenoid route involving the methyl-*D*-erythritol-4-phosphate (MEP) pathway [100,101] and not by the more common mevalonate pathway [101] (Figure 8).

Cyanobacteria can be free-living organisms in the water column (i.e., plankton existence) or grow attached to sediments (i.e., benthic populations). A report documented 41 different species/types of microorganisms capable of excreting geosmin and 2-MIB in diverse aquatic sources across the United States over a 22-year period [102]. This work could be broadened to survey similar aquatic systems across all states in the US. Chen et al. [103] measured these malodorous metabolites in benthic cyanobacterial mats using headspace SPME-GC-MS. To collect the headspace gas, a unique two column system was employed, one inserted head-down over the benthic algal mat and the other column over an area without the microalgae, to serve as the negative control. Although the types of cyanobacteria populating these mats are not known, the two column method provides a fairly simple and inexpensive technique for measuring the emission rates of these malodorants and other VOCs as well [103]. A SPME-GC-MS approach was employed for monitoring geosmin and 2-MIB in the waters of Glenmore reservoir (Calgary, AB, Canada), which had experienced periodic outbreaks of T&O issues [104]. Chrysophytes (golden-brown/golden microalgae) and diatoms (*Uroglena Americana*, *Dinobryon* spp., *Synura petersenii*, and *Asterionella formosa*) were responsible for these VOCs.

**Figure 9:**
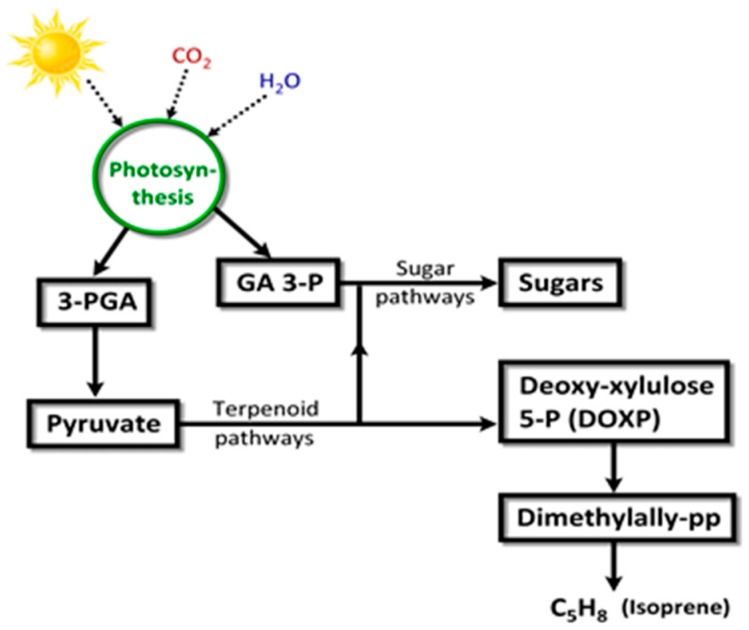
Isoprene production by the DOXP pathway. 3-PGA, 3-phosphoglyceric acid or glycerate 3-phosphate; GA 3-P, glyceraldehyde 3-phosphate. Adapted from [106,108] with permission.

Isoprene (2-methyl-1,3-butadiene; C_5_H_8_; CH_2_=C(CH_3_)-CH=CH_2_) is a colorless, low boiling point (~34 °C) VOC produced by members of the Kingdom Plantae, to which several microalgae also belong. Isoprene is also an abundant VOC in human breath [105]. Isoprene, a major BVOC (and AVOC), is biosynthesized by two different pathways: (a) cytosolic mevalonate pathway (Figure 8); (b) chloroplastic, photosynthetic, 1-deoxy-d-xylulose-5-phosphate (DOXP) pathway [106] (Figure 9). In algae, carbon partition to isoprene goes through the DOXP pathway. Isoprene is produced during photosynthesis, in the presence of sunlight, CO_2_, and H_2_O, via terpenoid pathways to DOXP [106,107].

Isoprene can be oxidized by hydroxyl radicals in the atmosphere to generate hydroperoxides which can then react with nitric oxide (NO) to form nitrogen dioxide (NO_2_); (NO and NO_2_ are referred together as NO_x_). Sunlight causes the photolysis of NO_2_ to generate ozone (O_3_) [109], as shown below.

RH + OH + O_2_ → RO_2_ + H_2_O

RO_2_ + NO → RO + NO_2_

2(NO_2_ + O_2_ → NO + O_3_)

An undesirable effect of isoprene is to increase the residence time of GHGs in the troposphere. Isoprene also influences SOA generation and thereby impacts global climate changes, similar to methane. Isoprene is an infochemical (or semiochemical) in that it deters plant predators by attracting predators of the predator, i.e., herbivores [110]. Given these complex functions of isoprene, it is important to note that marine isoprene production was estimated to be between 0.1 and 1.9 teragram (Tg) carbon/year [111], and that it might even be as high as ~12 Tg C/year [112].

Bonsang et al. [113] first presented evidence for a biotic origin of isoprene in a marine environment. Subsequently, using ocean samples as well as lab cultures, several phytoplankton were inferred or actually shown, to emit isoprene [114,115]. These emissions could affect cloud properties [116]. Diatoms, *Nitzschia* sp., *Porosira glacialis*, and *Odontella mobiliensis* culture headspace was sampled for a period of three weeks using the purge and trap (P&T) method, and the non-methane hydrocarbons (NMHC) were collected and analyzed by GC-MS. A number of NMHCs were produced by all cultures with isoprene being the major VOC with all the phytoplanktons [115]. It was concluded that since terrestrial plants and trees are remote from the marine surface atmosphere, and given the relatively short lifetime of isoprene (~2 h at noon sunlight), terrestrial isoprene emissions were unlikely to impact the ocean atmosphere. On the other hand, marine sources of isoprene (such as microalgae) could “significantly influence marine atmospheric chemistry” [115].

Several phytoplanktons (diatoms such as Phaeodactylum tricornutum, Thallassiosira weissflogii, and Chaetoceros affinis; coccolithophores such as *Emiliania huxleyi*; dinoflagellates like *Amphidinium operculatum*; and other microalgae such as *Prymnesium parvum* and *Synechococcus* sp.) monocultures were found to emit isoprene during the exponential phase of the microalgae’s growth (1 to 7 pmol/10^6^ cells/day). Isoprene levels declined in senescent cultures [111]. The accumulated headspace over the actively growing cultures was sampled and analyzed using GC-FID [111]. Since bacterial source for isoprene would be expected to peak during microalgal senescence due to feeding on dead/dying phytoplankton, the authors excluded biopollutant or contamination as a source of the measured isoprene, although axenic cultures of the algae would have removed all doubt. The authors acknowledge this possibility by stating, “While reasonable precaution was made and appropriate sterile techniques used to ensure their non-contamination with bacterial species during handling, the possibility of the presence of bacterial cells in these cultures must be allowed” [111].

Isoprene production rates were reported for ~30 strains of microalgae from seven different algal classes: Bacillariophyceae, Prymnesiophyceae, Dinophyceae, Cyanophyceae, Chlorophyceae, Cryptophyceae, and Prasinophyceae [112]. A total of 21 strains from across the seven microalgal classes were incubated without shaking, for a consistent period of 4 h light cycle, and then subjected to a purge of low-hydrocarbon containing compressed air. Gas from this purge was concentrated in a cryotrap kept at −160 °C. Isoprene in the concentrate was quantified using GC-FID. It was observed that isoprene production rates varied by nearly 2 orders of magnitude between the strains (0.03 to 1.3 μmol/h) [112]. In a different approach, Shaw et al. [117] used both axenic and xenic cultures of cyanobacteria and other phytoplanktons to monitor isoprene and NMHC emissions. The phytoplankton cultures examined were, *Prochlorococcus* (axenic and xenic), *Synechococcus* (axenic), *Emiliania huxleyi*, *Micromas pusilla* (flagellate, photosynthetic, picoeukaryotic cells, <3 μm, belonging to Prasinophyceae class of green algae), and *Pelagomonas calceolate* (uniflagellate, photosynthetic, ultra-planktonic marine alga). Since the cultures were axenic and xenic, bacterial contamination was continuously monitored and confirmed that isoprene production was independent of biopollutants. Culture headspace P&T was used to collect the volatiles which were analyzed by GC-FID. Although the culture incubation temperature was different (unique to the species), all cultures were under 14 h light (~110 μE/m^2^/*s*), and 10 h dark cycle. Isoprene production was related to light intensity and temperature effects. All species produced isoprene during exponential growth phase at rates of 1 × 10^−21^ to 4 × 10^−19^ mol/cell/day, depending on the species. This short list of phytoplankton emitting isoprene was enlarged by the authors with an examination of ~30 microalgal cultures, all of which were found to emit isoprene and monoterpenes. Rates of emission varied as above with species, chlorophyll concentration, light, temperature, and other parameters [118].

Isoprene emission from microalgae was correlated with light intensity. For example, there was a rapid increase in isoprene production by three diatom strains at low irradiance levels (<150 μmol/m^2^/s), accompanied by a gradual increase at higher irradiances (>250 μmol/m^2^/s) [119]. The phytoplanktons were *Thalassiosira weisflogii*, *Thalassiosira pseudonana*, *Chaetoceros neogracile*, and *Emiliania huxleyi*. All microalgae were grown at 22 °C without bacterial contamination by applying sterile techniques. After 7 to 14 days in culture, headspace was sampled for up to 8 h, and analyzed by GC-photoionization detector (PID) [119]. These authors also examined six microalgal monocultures [120]: *Thalassiosira weissflogii*; *Thalassasiosira pseudonana*; *Pleurochrysis carterae* (unicellular coccolithophore); *Karenia brevis* (marine dinoflagellate); *Prorocentrum minimum*; and *Rhodomonas salina* (Cryptophyta). Headspace VOCs were concentrated in a wet trap followed by a sorbent trap, and then analyzed using GC-MS. The microalgae had been subjected to 12 h of light stress (90–900 μmol/m^2^/s) followed by 12 h of dark prior to VOCs collection. Temperature stress was by incubation between 18 to 30 °C. Diatoms were the largest emitters of isoprene in this study. Other VOCs emitted included α-pinene, β-pinene, d-limonene, and camphene [120].

Generally, VOC emissions increased rapidly under low light, and gradually increased under stronger light intensities [119]. Compared to isoprene, the emissions of monoterpene were an order of magnitude lower at all light and temperature regimens. It was concluded that isoprene emissions from the various phytoplankton could play a role in SOA formation and thereby modulate marine cloud properties, eventually influencing the climate [120]. Carbon cost of headspace isoprene emissions in diatoms (*Phaeodactylum tricornutum* and *Chaetoceros calcitrans*) was calculated as being ~2000-fold lower relative to terrestrial plants as a fraction of photosynthesis. It was therefore concluded that marine phytoplankton’s contribution to atmospheric carbon levels might be significantly higher than previously estimated [121] relative to terrestrial plants. Since marine phytoplankton (for most part) tend to emit either DMS or isoprene, and further since these two volatiles had opposite latitudinal gradients, it was suggested that DMS emission was dominant in the polar waters whereas isoprene emissions were higher in tropical (warmer) oceans. Global warming may however “expand the geographic range of marine isoprene-emitters” [122].

**Figure 10:**
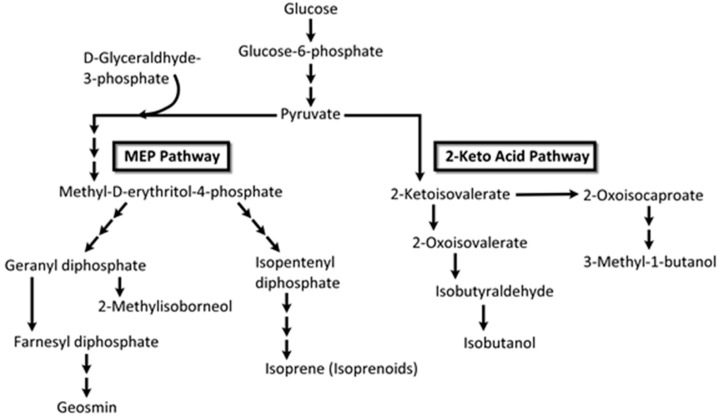
Interconnectivity of metabolic pathways. MEP, methyl-d-erythritol-4-phosphate. Regardless of metabolism, the biosynthesis of VOCs occurs through the formation of pyruvate.

Besides isoprene, diverse classes of other VOCs are also produced by microalgae with biological and ecological significance. Such VOC classes include alcohols, esters, aldehydes, hydrocarbons, terpene derivatives, ketones, furans, carboxylic acids, fatty acids, carotenoid derivatives, and sulfur compounds [36,123]. The biochemical pathways for some of these VOCs show connectivity between primary and secondary metabolic pathways [123] (Figure 10).

**Figure 11:**
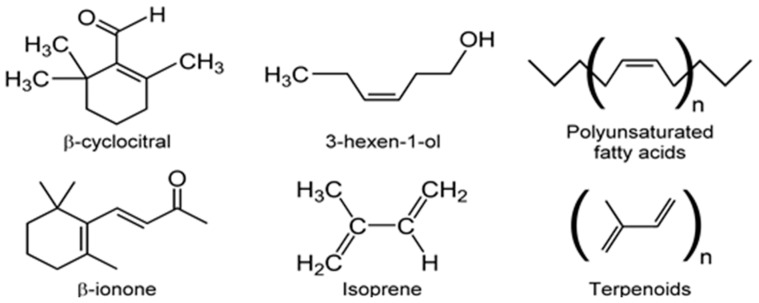
Chemical structures of a few prominent microalgal (‘other’) VOCs.

For example, nor-carotenoids are derived from the oxidative cleavage of carotenes and xanthophylls. The degradation products of these reactions generate a variety of VOCs such as α-ionone, β-ionone, α-cyclocitral, and β-cyclocitral (Figure 11) [36,98]. Volatile fatty acids (VFAs) are direct or indirect products of lipid metabolism. In addition to geosmin and 2-MIB, several ketones and ionones [99], as well as odorous compounds such as C_7_–C_10_ alkenes, 2,4-heptadienal, 2,4,7-octatriene, 2,4-decadienal, and 2,4,7-decatrienal are all emitted by microalgae (e.g., *Chrysophytes*, golden-brown or golden microalgae and diatoms) [104]. Despite considerable diversity of microalgae and the importance of microalgal VOC emissions, there is little quantitative information on VOCs biogeneration in vivo [86,123]. These observations should prompt quantitative investigations of VOCs biochemistry and consequent environmental impact.

Microalgae growing in natural marine environments were identified as major producers of VOCs including sulfate aerosols, isoprene, and monoterpenes [124]. Unpublished data identified 45 different VOCs being emitted by marine and freshwater algal cultures, although it was not clear whether the emitting species were microalgae or macro [124]. Walsh et al. [125] found *Microcystis aeruginosa* released copious amounts of VOCs including C_15_–C_21_ aliphatic hydrocarbons, naphthalene, terpenoids, β-cyclocitral, and β-ionone. Exposure to sunlight and varying the iron concentration of the culture medium affected both the specific VOCs emitted and their relative concentrations [125]. The authors used closed loop stripping analysis (CLSA) for the capture of VOCs. It was not clear whether the microalgae survived CLSA treatment. Chlorophyll *a*, a key component of phototrophic microalgae, correlated with the generation of VHCs such as chloroform and dichloromethane [126]. Capture and analysis of VOCs was by SPME-GC-MS. Wind speed and water temperature, along with seasonal variations, influenced VOCs production. In a rare example of computational analysis [127], the authors used partial least squares (PLS) modeling to highlight microalgal VOC emissions in the spring, whereas macroalgae emissions dominated at other times [126].

In the ocean, microalgae cause a rapid turnover of methanol within a timeframe of 1–7 days [128]. A P&T method was used to trap headspace gas of different marine phytoplankton in culture, including the cyanobacteria, *Synechococcus* spp., *Trichodesmium erythraeum*, *Prochlorococcus marinus* and the eukaryotic diatom, *Phaeodactylum tricornutum*, *Emiliania huxleyi*, the cryptophyte *Rhodomonas salina*, and the non-diatom heterokont, *Nannochloropsis oculata*. All microalgae cultures were axenic, except (emphasis added) *T. erythraeum*, which the authors noted was “notoriously difficult to maintain pure”. Headspace gas was analyzed by GC-MS. All microalgae produced methanol in concentrations ranging from 0.8 to 13.7 μM in culture. Methanol production was maximal near the early stationary phase [128]. In a similar study, methanol production was examined in the headspace of microalgae cultures including coccolithophore, dinoflagellates, a haptophyte, and a cyanobacterium. Production of micromolar quantities of methanol were estimated from these phytoplanktons during exponential and stationary growth phases. Certain similarities were observed between the production profiles of methanol and isoprene in the headspace from the eukaryotic dinoflagellate, *Amphidinium operculatum* [129]. The conclusions from these studies were that the phytoplanktons have the potential to contribute significant amounts of methanol to ocean waters and then to the atmosphere. Since marine phytoplankton are the most abundant organisms, their methanol production could rival those of terrestrial biotic sources of methanol [128], since even small amounts of methanol produced could turn into a large reservoir due to the sheer population density of the phytoplankton [129].

Five marine phytoplankton species—*Calcidiscus leptoporous* (coccolithophore), *Emiliania huxleyi*, *Phaeodactylum tricornutum*, *Chaetoceros neogracilis*, and *Dunaliella tertiolecta*—belonging to three different microalgal classes and occurring in coastal and open ocean waters, were studied using headspace gas analysis by GC-MS [130]. All cultures were axenic except *C. leptoporous*. The cultures were grown at 20–25 °C (depending on species) in an even split cycle of 12 h of light (~250 μE/s/m^2^) and 12 h dark. VOC samplings were performed in the middle of the light cycle, over 3–4 days. The strongest emitters of methyl bromide were *C. neogracilis* and *P. tricornutum*; *C. neogracilis* was also the strongest emitter of isoprene. Other VOCs emitted by all phytoplanktons were chloroform, dichloromethane, trichloroethylene, chlorobenzene, tetrachloroethylene, and dichlorobenzene, along with 1,1-dichloroethane and 1,2-dichloroethane. With all species, trichloroethene production was more significant than tetrachloroethene. The volatile bouquet included VHCs and NMHCs [130].

**Figure 12:**
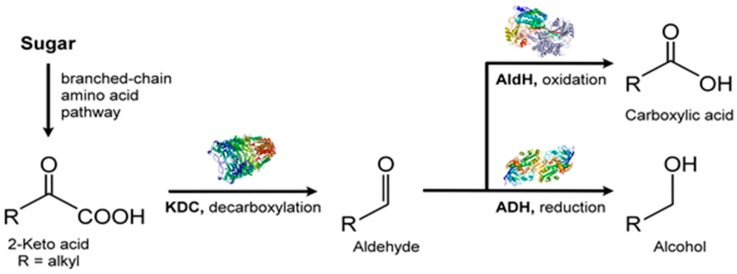
2-Ketoacid pathway for the production of alcohols and acids. KDC, 2-ketoacid decarboxylase; ADH, alcohol dehydrogenase; AldH, aldehyde dehydrogenase.

VOC profiles of the heterotrophic cyanobacterium, *Phormidium autumnale*, were studied using axenic cultures in a bubble column growth bioreactor at 25 °C, pH 7.6 [131]. As above, light and dark cycles were evenly split in 12 h portions and the light intensity was 15 μmol/s/m^2^. Headspace gas was sampled using SPME and the VOCs were analyzed by GC-MS. A total of 68 VOCs were identified during this study, with 3-methyl-butanol being the major volatile, at a concentration of ~142 μg/mg dry weight of culture [131]. Several VOCs belonged to the terpenoid class of volatiles and included β-ionone, β-cyclocitral (Figure 11) and 5,6-epoxy-β-ionone, hexanol, and hexanal, along with VOCs from the 2-keto acid pathway such as 3-methyl-butanol, propanol, and butanol (Figure 12).

Amongst the most abundant VOCs were ketones, such as 2,3-butanedione and dehydro-2-methyl-furanone. Aldehydes were fewer in the VOC profile and were mostly C_2_ and C_4_ compounds. Only linear short chain (C_3_–C_5_) alcohols appeared in the headspace gas and such alcohols are characteristic biomarkers for the cyanobacteria. With acidic volatiles, acetic acid dominated followed by butanoic acid, isobutyric acid, and isovaleric acid. This analysis of the volatile profile, while not entirely comprehensive, is nevertheless a good start to VOC metabolome of in vivo microalgae.

Methane is one of the most potent GHGs and second in importance only to CO_2_. Consequently, methane plays an important role in atmospheric chemistry [132]. The GWP of methane is 28 since it absorbs more energy than CO_2_ and biogenic emissions of methane play an important role in this phenomenon vis-à-vis even fossil-fuel industry [133]. Therefore, methane emissions from monoclonal cultures of *Emiliania huxleyi* were a significant finding [134]. The microalgae were grown at 20 °C inside a closed flask with ~500 mL headspace. Cells were grown on a day–night cycle (~450 μE over 10 days) of 16 h and 8 h, respectively. Headspace was sampled between days 4 and 10 of growth and GC/isotope ratio-MS was used to quantify methane [134]. Methane production was too low to quantitate during exponential growth (day 7). It was estimated that thereafter methane production rate ranged anywhere between 1/10 to 10-fold greater, relative to methane production by terrestrial plants. Due to its widespread distribution in the marine environment, *Emiliania huxleyi* might contribute significantly to methane oversaturation of ocean surface waters [134]. Some researchers have proposed increasing algae growth for cattle feed, as inclusion of algae in the feed may decrease the animal’s methane emissions. However, it is important that any decrease in methane emission from cattle is not offset by the VOCs generated from large-scale cultivation of algae.

In another rarity amongst in vivo microalgae VOC profile characterization, Durme et al. [135] used computational algorithms and pattern recognition software to segregate microalgae on the basis of their VOC profiles. Five species of microalgae were investigated: *Botryococcus braunii* (green, pyramid-shaped planktonic microalgae)*, Rhodomonas Sp.*, *Tetraselmis* sp., *Nannochloropsis oculata*, and *Chlorella vulgaris*. A variety of VOC classes were identified in the headspace gas which was analyzed using SPME-GC-MS. The VOC classes included VOSCs, linear, branched, and aromatic aldehydes, alcohols, ketones, terpenes, norisoprenoids, esters, acids, and furans [135]. All microorganisms emitted high levels of sulfur compounds such as DMDS, DMTS, and methanethiol, along with diketones, α-ionone, β-ionone, and aldehydes such as 2,4-alkadienals and 2,4,6-alkatrienals. Principal component analysis (PCA) of the VOC bouquet segregated the microalgae according to species [135]. In another study, headspace VOCs from the cyanobacteria, *Spirulina platensis*, *Nostoc* spp., and *Anabaena* spp. (filamentous, planktonic, cyanobacterium) were analyzed using GC-MS. Geosmin was not detected in any the three cyanobacteria, although 2-MIB was present in all emissions. Amongst a total of 17 VOCs identified, the main VOCs were medium chain length alkanes, 2-pentyl-furan, β-cyclocitral, and β-ionone [136]. These authors also performed PCA, along with hierarchical cluster analysis (HCA), to segregate microalgal species based on VOC profiles [136]. Such pattern recognition tools must play a key role in the analysis of microalgal VOCs and microalgal metobolome.

## 4. Functions of Microalgal VOCs

Thus far, several VOCs were identified along with pathways for the production of microalgal VOCs, and theories regarding their roles in ecology, atmospheric chemistry, and global climate. But what of the microalgae itself? What is the purpose behind the production of VOCs by the microalgae? VOC emissions reduce carbon availability for the microalgae’s growth, survival, development, and reproduction. So, how does the loss of crucial carbon reservoir benefit the organism? Why should microalgae undertake energy-expensive processes such as the production and release of these VOCs? There are no easy answers to these questions. In some cases, VOCs may be produced as a detoxification mechanism, as with the formation of Se-VOCs. Elsewhere, Se-VOCs might have a beneficial effect on microalgae’s growth and development. For example, with *Emiliania huxleyi* [137], it was found that the microalgae converted Se to Se-VOCs, and utilized the latter for the synthesis of selenoproteins. The production of N_2_O might involve nitrate assimilation, regulated through NO.

Throughout this review, several references were made regarding one or more VOC levels changing due to the microalgae’s growth status (log phase, stationary phase, senescence). Whether such fluctuations in VOC levels are causally related to growth or serendipitous is unclear. Some metabolites were identified with osmolytic function, though even this idea is not unequivocally accepted [138]. In the marine environment, there is an excess of sulfur and a relative shortage of N_2_, which is critical for phytoplankton growth. Therefore, it has been argued that DMSP production may be regarded as an ‘overflow mechanism’ whereby the microalgae balances its nutrients and adapts to shifting environmental situations. Thus, DMSP lyase activity is a regulatory mechanism and the release of DMS might be incidental toward maintaining the DMSP equilibrium [138].

Metabolites excreted by cyanobacteria (geosmin, 2-MIB, β-ionone, β-cyclocitral), purchased as purified compounds inhibited the growth of the eukaryotic green alga, *Chlorella pyrenoidosa*. Other VOCs showing similar growth inhibitory properties included monoterpene alcohols, straight chain fatty acids (C_7_–C_12_), and straight chain alcohols and aldehydes, leading to the conclusion that these VOCs might act as allelopathic agents towards the phytoplankton [139]. However, the concentrations (10 mg/mL) at which these VOCs displayed growth inhibitory properties were high, leaving the physiological relevance ambiguous. In conclusion, VOC emissions of microalgae have included postulates of defense mechanism, stress response, intra- and inter-species communication, and regulating prey–predator interactions. As noted earlier, CNBr emission was part of a daily “housekeeping” activity by the microalgae that kept the environment free of competitors [93].

### Do Microalgae Communicate?

Due to paucity of data on the physiological significance of microalgal VOC emissions, there is a need to focus on VOC emissions from plants for drawing parallels and “lessons learned” [140]. Plants are relevant since several photosynthetic microalgae belong to the Kingdom Plantae. Plants respond to neighbor’s arrival, mechanical wounding, herbivore feeding, or to pathogen infections, by releasing VOCs, leading to the characterization of such events as “plant–plant communication” or “talking trees” and “plant’s cry for help” [141,142]. The term “plant volatilome” was proposed by Maffei et al. [143], and has since been widely used [144,145]. The photosynthetic behavior of microalgae resembles that of plants. As such, plant and microalgae VOC emissions might occur under the influence of similar factors (Figure 2) [146,147]. Furthermore, VOC chemical structures and even functions in the plants might be replicated in the microalgae [148]. Thus, VOCs may play the role of infochemicals in the microalgae, constituting a unique ‘vocabulary’ for communicating within and across species. Such a scenario is plausible, since VOCs can travel far from the point of source with the capacity to traverse subterranean, land, water, and air due to their low-to-moderate hydrophilicity, ability to dissolve in water, or disperse at the air–water interphase [149].

Infochemicals are non-verbal (non-acoustic) communications involving chemicals containing actual information (‘cues’) or precursors to information (‘signal’). Infochemicals are classified as ‘pheromones’ (generally, intra-species communication) and allelochemicals (generally, inter-species communication) [150]. Semiochemicals are communication cues which may be inter-species or intra-species. For example, microalgal kairomones act as chemical cues to predators, enabling them to seek out prey and thus are not beneficial to the microalgae. Allelochemicals are secondary metabolites (such as VOCs) which may have a detrimental effect on a species different from the emitting organism (negative allelopathy) or a beneficial effect (positive allelopathy). Certain kairomones exemplify negative allelopathy. Such VOC-mediated communications are not limited to microalgae, and have been known to occur in a number of different organisms, ranging from bacteria [148,151,152], fungi [149,152], to higher organisms such as plants [141,142,153,154], insects [155,156,157,158], and animals [159].

**Figure 13:**
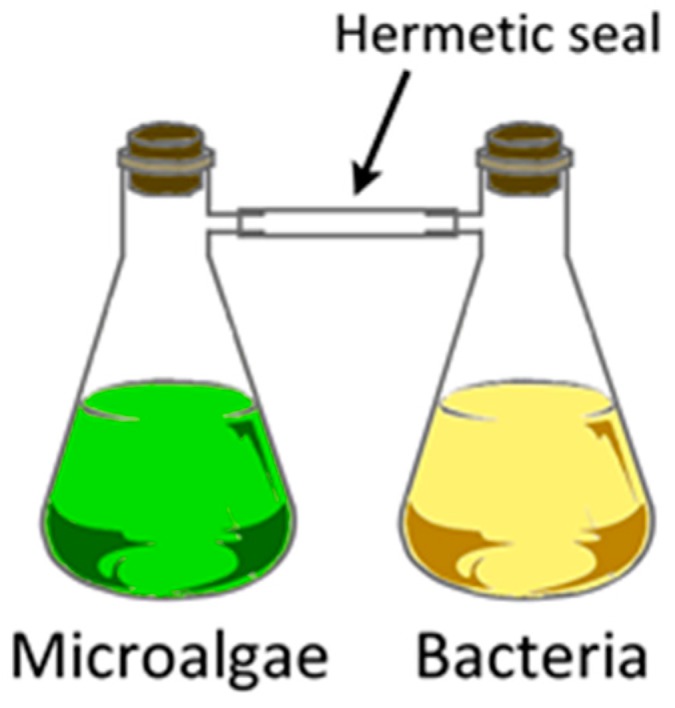
Schematic of an experimental set-up for intra- and inter-species continuous communication. Figure adapted from [161] with permission.

Intra-species communication via VOCs was demonstrated by exposing ‘normal’ *Chlamydomonas reinhardtii* cells to gases collected from the headspace of ‘salt-stressed’ (NaCl, 300 mM; Na_2_CO_3_, 150 mM) cells which resulted in growth inhibition of the normal cells. Besides headspace volatiles, there was no other means of contact between the two types of cells [160]. This ‘non-contact inhibition’ was accomplished by growing the salt-stressed cells in a flask that was connected to a second flask such that only VOCs from the former flask were introduced into the latter (Figure 13). A total of 33 VOCs were identified from the headspace using thermal desorption (TD)-GC-MS, including 1-octene, 3,4-dimethyl hexane, octanal, hexanal, nonane, 1-decene, undecane, nonanal, dodecane, with hexanal being the most abundant VOC from NaCl stressed cells, whereas 3,4-dimethyl hexane and 5-methyl-2-heptene were released in copious amounts from Na_2_CO_3_-stressed *Chlamydomonas reinhardtii* [160]. A caveat to the system is the gas-tight connection between the two flasks with the potential for anaerobic processes setting in. Consequently, growth needs to be monitored carefully.

The authors expanded these observations to *inter-species* non-contact communication as well [160]. *Microcystis flos-aquae* (freshwater cyanobacteria) cells were grown in medium and kept in a light (16 h, 50 μmol/m^2^/s, illumination intensity) and dark (8 h) cycle at 23 °C. Cells were in mid-logarithmic growth phase when VOCs were collected by dynamic headspace air-circulation method [160]. Numerous VOCs were identified under various classes such as furans, sulfur compounds, terpenoids, benzenes, aldehydes, esters, and hydrocarbons. When *Chlorella vulgaris* cells were exposed to VOCs emitted by *M. flos-aquae* under non-nitrogen growth conditions, cell propagation and photosynthetic pigment declined in the former, indicating a negative allelochemical (toxic) effect of the *M. flos-aquae* VOCs [162]. An interesting example of inter-species communication with the opposite effect, i.e., whereby VOCs emitted by bacteria had a positive effect upon microalgae was reported [161]. Nearly 45 VOCs including 2,3-butanediol, acetoin (3-hydroxy-2-butanone) were emitted by *Azospirillum brasilense* (nitrogen fixing Gram-negative bacteria) and *Bacillus pumilus* (aerobic, spore forming Gram-positive bacteria). These bacterial headspace VOCs were exposed to a microalgal culture of *Chlorella sorokiniana* (freshwater green microalgae) in a non-contact fashion, after taking the precaution to scrub the headspace VOCs free of CO_2_. Exposing the microalgae to bacterial VOCs led to a six-fold increase in growth and an increase in the cell volume by approximately three-fold. This was an example of beneficial (positive allelochemical) effects of the bacterial VOCs upon microalgae, by promoting growth [161].

**Figure 14:**
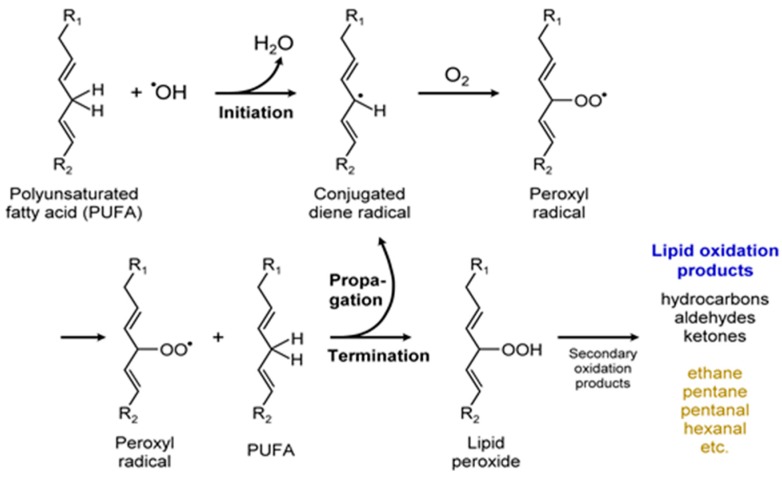
Volatile products of polyunsaturated fatty acid and lipid oxidation.

Certain polyunsaturated fatty acids (PUFA) were identified as allelopathic agents in microalgae [36]. The production of several different volatile products from PUFAs—including hydrocarbons, aldehydes, and ketones—are shown in Figure 14. As with any other metabolite, production of volatile short chain fatty acids (SCFAs) and PUFA depend on the various factors influencing the growth and development of the microalgae (Figure 2). In a different example, this one involving a pheromone-like property, fatty acid derived cyclic and acyclic hydrocarbons were proposed as sex pheromones for sperm attraction in marine microalgae. Similar or same VOCs were also identified in freshwater diatoms; however, their functions in diatoms are unknown [97]. The chemistry of a particular VOC, timing of its release, persistence, and diffusivity in the environment, are all critical to semiochemical activity [36]. Thus, VOC allelopathy (positive or negative) sheds light on microalgal community structure, population dynamics, defense mechanism, and might provide the microalgae with a competitive advantage [32], or sometimes act to the disadvantage of the organism by promoting predator attack, aiding the enemy. Finally, abundant production of PUFA by microalgae is of commercial interest due to their potential health benefits and non-nutritional applications such as in the manufacture of paints and varnishes.

## 5. Presymptomatic Diagnostics—A Potential VOC Application?

### 5.1. Presymptomatic Diagnostics

Presymptomatic diagnostics is an important goal in human health [163]. It is important to distinguish between asymptomatic individuals from uninfected individuals (healthy cohorts). Is an individual uninfected, or infected by a pathogen but the disease has not progressed to the extent that symptoms appear, or infected but not displaying symptoms? Extrapolating to microalgal cultivation, an intriguing application of VOC technology is in the early detection of algal “pond crash” due to predator attack [33,34,35]. The VOC technology platform could thus be used to distinguish individuals, whether microalgae or humans, who are uninfected or infected but not displaying symptoms, or infected but infection progression is insufficient to display symptoms. It is estimated that pond crash is responsible for the failure of nearly one-third of all annualized algae production. The situation is exacerbated by the fact that current diagnostic tests for algal pond crashes involve laborious techniques such as flow cytometry or molecular biotechnology, which require complex procedures that are expensive, not-portable, posing difficulties for handling multiple specimens, not amenable to automation, unsuited for rapid field tests, offering poor overall algal pond coverage, and requiring bench-top equipment and trained personnel to run the tests and correctly interpret the results [33]. Could VOCs be reliable biomarkers for microalgae’s SoH?

### 5.2. VOCs for Presymptomatic Diagnostics

A revolutionary approach could be to utilize VOCs to predict healthy microalgal ponds and presymptomatically detect predator attack in ORPs or PBRs. There are several advantages to presymptomatic diagnostics including time for instituting remedial measures, containment of outbreak, forecasting pond crashes, and decreasing cost. Successful demonstration of VOC-based presymptomatic diagnostics could also broaden the scope to macroalgal cultivation. There are tantalizing publications in the literature for promptly initiating this approach. For example, breath VOC analysis using Monte Carlo simulation identified influenza-virus specific biomarkers in individuals who had previously been vaccinated with live attenuated influenza virus (LAIV) [164]. The study authors concluded that “a breath test for these VOCs could potentially identify humans who are acutely infected with influenza, but *who have not yet developed clinical symptoms or signs of disease*” (emphasis added) [164]. The VOC signatures could be used as ‘stand alone’ biomarkers of pond crash, or in combination with other tools/techniques for improving prediction capabilities. For example, conventional tools for predicting asthma in children yielded poor positive predictive values (~60%). Adding exhaled breath VOCs detection and gene expression biomarkers, classified 89% of the children accurately, a significant improvement in disease predictability [165].

### 5.3. VOC Metabolomics (Volatilomics)

Several different types of VOCs might be expected from microalgae in vivo. These include VOCs produced by the microalgae, VOCs from interactions between microalgae and a specific growth medium; background VOCs from the marine environment, growth medium, or ambient air; and VOCs due to the presence of biological pollutants/predators. Absence of an analyte or a group of analytes in the VOC profile might be just as distinctive and discriminatory. A combined strategy of specific and general VOC emission analyses can dramatically increase diagnostic specificity and sensitivity. The goal is to establish a definitive link between a particular VOC, groups of VOCs, absence of certain VOCs, and/or emission patterns of VOCs, either individually or in some combination, to the microalgae’s SoH.

The approach towards using VOC technology for presymptomatic algal pond crash prediction and early pond crash forensics could include VOCs specific to the microalgae that is being cultivated, qualitative and/or quantitative deviations from such VOC levels, VOCs specific to the predator suspected of invading the algal pond, qualitative and/or quantitative deviations from such predator VOCs levels, and pattern recognition [127] for the analysis of the microalgae and/or the predator VOC profiles. This “volatilome” [12,13,14,15,16,17,143,144,145] could be a ‘stand alone’ resource for making robust predictions of presymptomatic pond crashes, or complement/supplement one or more traditional ‘omics’ technologies [166]. Algal pond crash-causing predators include microorganisms such as bacteria. It is therefore especially relevant that VOCs have already been used for bacterial identification, even in mixed/co-cultures, down to species and serovar levels [167,168,169,170,171]. There are also publications on VOC-mediated interspecies communications (vide supra) including predator–prey interactions involving protists [172] (phytoplankton predators). These studies have been enlarged by the availability of metabolomic and volatilomic databases of humans, bacteria, and algae. Specific to VOCs, there are metabolomic/volatilomic databases and resources such as “VocBinBase” [173], flavornet, http://www.flavornet.org/, “mVOC”, database of microbial volatiles [174], “ABA-Cloud” [175], “MetaboLights” (http://www.ebi.ac.uk/metabolights/) [176], “DOVE-MO” (Kalderas, J. “DOVE-MO” database of volatiles emitted by microorganisms. *Diploma Thesis*, *University of Rostock*, *Germany*, 2011; cited in [149]), databases and software packages [30,144,177,178,179], and National Institute of Standards and Technology (NIST), Standard Reference Database, https://www.nist.gov/srd/nist-standard-reference-database-1a-v14, all of which may be consulted for VOC identification, matching metabolites to specific metabolic pathways [180], and building a volatilome unique to microalgae and/or predator, to facilitate presymptomatic pond crash forensics. Such studies will be enabled by a knowledge of VOCs linked to metabolic processes (Table 2).

### 5.4. VOCs during Growth/Senescence

In order to realize presymptomatic pond crash forensics, it is essential to establish a definitive role for VOCs in microalgae physiology, growth, and development. In other words, is there a link between VOC secondary metabolites emission that is correlated with growth, senescence, or death of the microorganism? Throughout this review, several papers were summarized that were suggestive of a positive relationship between the microalgae’s SoH and specific VOC emissions. These observations are briefly revisited here along with new data not discussed previously.

Haas [52] was perhaps the first to scientifically document a relationship between decomposing seaweed and VOSCs, albeit in macroalgae. Similar correlations between VOSC and microalgae’s death or decay were published later. For example, when microalgae aged or were consumed by predators, DMS was released into the water column environment [59]. Decomposing blue-green algal mats released copious amounts of VOSCs such as methylmercaptan and H_2_S [69]. Other odorous volatiles like geosmin and 2-MIB were shown to be released by phytoplankton during cell lysis [104], perhaps under predator attack. Indeed, DMS was released in order to deter predators [64,65]. Florez-Leiva et al. [42] demonstrated high emissions of N_2_O in the headspace of *Nannochloris* when the microalgae were undergoing senescence. On the other hand, they also noted that methane emission increased steadily between 2 to 10 μmol/m^3^ during the growth phase of this phytoplankton [42]. Isoprene was proposed as a biomarker for senescence due to a decrease in its levels in senescent cultures of several microalgae [111]. Methanol production was maximal during early stationary phase for a range of phytoplankton including coccolithophore, dinoflagellate, haptophyte, and cyanobacteria [128], whilst chloroform levels were similarly maximal during logarithmic growth phase of the unicellular red alga, *Porphyridium purpurem* [90]. Abundant acetic acid production was observed from cyanobacterium *Phormidium autumnale* [131] and this was linked to programmed cell death (PCD) of the microalgae. Thus, *Chlamydomonas reinhardtii*, a unicellular green microalga, underwent PCD in the presence of acetic acid along with the emission of abundant quantities of >30 different VOCs released into the headspace [181]. Interestingly, VOCs released during PCD also inhibited the growth of normal microalga, acting as infochemicals (negative allelochemicals) [181].

Under normal, healthy operating conditions, algal ponds will be aerobic and low emissions of VOCs are expected. During catastrophic failure of the culture, algal ponds undergo a transition to an anaerobic condition, and a variety of nitrogen and sulfur VOCs are emitted along with methane [124]; however, the anaerobic requirement has been challenged [182]. As noted above, T&O compounds may be released during senescence or cell lysis [36]. Heightened release of terpenoid VOCs, like 2-methyl-1,3-butadiene, may occur during the stationary or senescence phases; however, little research has been conducted on these biological activities [36]. Contrary to an earlier report [104], geosmin was suggested as an indicator of live cells of *Nostoc flagelliforme* (cyanobacterium). Headspace samples were collected from this microalgae at defined intervals over 1 to 20 days and analyzed using SPME-GC-MS [183]. Eleven different VOCs were detected, amongst which geosmin levels declined, correlating with decreased cell viability, whereas indole levels increased simultaneously [183]. In agreement with previous reports, sulfur compounds such as DMS and DMTS (along with β-cyclocitral and β-ionone) were dominant during the decomposition phase, whereas 2-MIB was elevated during the growth phase [184]. Finally, Durme et al. [135] were able to segregate four different microalgae species using PCA of VOC profiles. Likewise, Milovanovic et al. [136] were able to separate different cyanobacterial species using PCA and HCA of VOC emissions. Furthermore, these authors were also able to correctly segregate two different samples of *Spirulina* using unique VOC emissions and pattern recognition algorithms [136].

Considered together, the foregoing implies that certain VOCs might be biomarkers of algal growth and/or death or senescence, or that some VOCs are produced in response to predators whilst others are indicative of healthy, exponential growth of the microalgae. There is also evidence that VOC profiles might be useful in identifying specific species of microalgae. Supporting this claim is the observation that C_3_–C_5_ alcohols might be characteristic biomarkers of cyanobacteria. Chance et al. [91] showed that iodide production was sensitive to strain level differences amongst the microalgae. It is perhaps important to note that not only specific VOCs, but also quantitative differences in VOC levels and VOC patterns, along with the absence of certain VOCs, will be collectively relevant for prognosticating regarding microalgae, predator, or pond health.

### 5.5. VOCs in Predator–Prey Interactions

The final consideration for presymptomatic diagnostics of microalgal pond crash after predator attack concerns the role of VOCs emission as a consequence of predator–prey interactions. Here too, there are intriguing publications suggesting a role for VOCs as infochemicals, either favoring or detrimental to the microalgae (kairomones). For instance, release of DMS under predator attack has been attributed to cell damage by grazing, leading to a mixing of the intracellularly-segregated DMSP and DMSP lyase, resulting in the release of volatile DMS [64,65,97]. Grazing might indeed be an important factor in the release of VOCs from microalgae which serve as deterrent to the grazer or as food finding cues to the predator. In a variation of this theme, short chain unsaturated aldehydes derived from oxylipin chemistry produced by marine diatoms, a major class of planktonic microalgae, were shown to induce reproductive failures in their natural predator, the copepod crustaceans. These failures included abortions, congenital malformation of offspring, and decreased larval growth, collectively contributing to securing the prey [185]. In a role reversal, VOCs produced by *Pseudonitzschia delicatissima* (planktonic diatom) and *Prorocentrum minimum* (dinoflagellate) attracted their predator, a zooplankton grazer, *Centropages typicus*, an example of negative allelopathy [186]. On the other hand, VOCs emitted by *Skeletonema marinoi* (another planktonic diatom) was favorable to the survival of the emitter by repelling the grazer and protecting the diatom [186]. It was concluded that these VOCs prompt chemokinesis according to the particular type of microalgal species and the concentration of the VOCs being produced [186].

Moelzner, Fink, and colleagues [1,187,188,189] demonstrated the role of VOCs in modulating prey-predator interactions. Since the chemical nature of the VOCs emitted as well as their abundance, depended upon a number of factors (Figure 2), it was interesting to note that the predator (freshwater snail) was attracted to VOCs released from nutrient-rich cultures of the microalgae, *Uronema* (formerly *Ulothrix*) *fimbriata* [187]. It was concluded that microalgal VOCs served as kairomones for aquatic herbivores. Release of VOCs upon mechanical wounding or predator grazing yielded a bouquet of volatiles that either attracted or repelled the predator. During grazing by gastropods, the radula (“rasping tongue”) of these animals scrapes over the microalgal mat which causes cell disruption, resulting in the release of volatiles. A VOC threshold concentration from *Uronema fimbriata*, was necessary to direct the foraging activity of the freshwater gastropod snail, *Lymnaea stagnalis*, towards the prey [188]. These data were expanded by using a synthetic mixture of specific VOCs that were commercially purchased to replicate the naturally observed infochemical activity. A mixture of 1-penten-3-one, 1-penten-3-ol, trans-2-pentenal, and 2,4-heptadienal, which were part of the VOCs emitted by the benthic green microalgae *Uronema fimbriata*, was able to attract the snails as foraging infochemicals [189]. Not only that, the grazers were able to distinguish between high and low quality food resources based on the odor quality of the VOCs [189]. The VOCs from axenic cultures of *Uronema* were extracted using CLSA and were sorbed onto a Tenax TA substrate. The VOCs were then thermally desorbed and analyzed by GC-MS. Thirteen different VOCs were identified in the desorbed mixture. Out of these, a synthetic VOC mixture was prepared using 1-penten-3-one, 1-penten-3-ol, 2-pentenal, and 2,4-heptadienal. This VOCs mixture acted as kairomones, benefitting the grazing, freshwater snail, *Radix ovata* [1]. The negative controls were a VOC mixture that omitted 2,4-heptadienal or used 2,4-heptadienal alone, both of which were ineffective in attracting the snail [1]. Despite these fascinating studies, to the best of our knowledge, there are no microalgal publications mirroring the plant’s “cry for help” [141], where VOCs released by the microalgae attracted predators of the microalgal predators.

### 5.6. Caveats to VOC-Based Presymptomatic Diagnostics

Admittedly, the results summarized above are not applicable for using VOCs to deter predator invasion in microalgal ponds. Rather these are biosignatures of prey under attack, signaling predator presence and enabling remedial actions to be implemented. There are several concerns that need to be addressed before volatilome-mediated pond crash forensics becomes a reality. First, successful launch of any diagnostic test depends on its sensitivity and specificity [190]. There may be a situation where the sensitivity of VOCs emitted by the microalgae or the emission patterns are not sufficiently specific to a microalgae or to a predator. This in turn depends at minimum upon the VOCs extraction capabilities from the matrix, concentration of the VOCs, resolution power of the GC system, and sensitivity of the VOC detector (vide infra). Specificity of detection could be impacted by environmental and ambient air VOC confounders [191]. There is the possibility that microalgal and/or predator-emitted VOCs, especially in ORPs, could get diluted quickly in ambient air and either dissipate entirely or become transformed into different VOC species due to abiotic reactions, resulting in misleading conclusions. With ORP culture, environmental factors such as wind, rain, and snow also need to be considered which can disperse the microalgal VOCs or interfere with sample collection. Other difficulties include an overlap, or lack thereof, of VOC signatures between microalgal species and the growth rate of microalgae which will affect VOCs production, collection, and analyses. Highly sensitive analytical solutions with parts-per-trillion (ppt) sensitivity might compensate for these shortcomings. Adequate positive and negative controls are critical not only during VOCs collection, but also during analysis, including standards for quantitation of emissions. Likewise, VOCs production by microalgae must be standardized based on biomass quantification, cell count, ash-free weight, lipids content, chlorophyll content, photosynthetic efficiency, etc.

## 6. Tools and Techniques for VOCs Collection and Detection

### 6.1. Microalgal Cultivation

**Figure 15:**
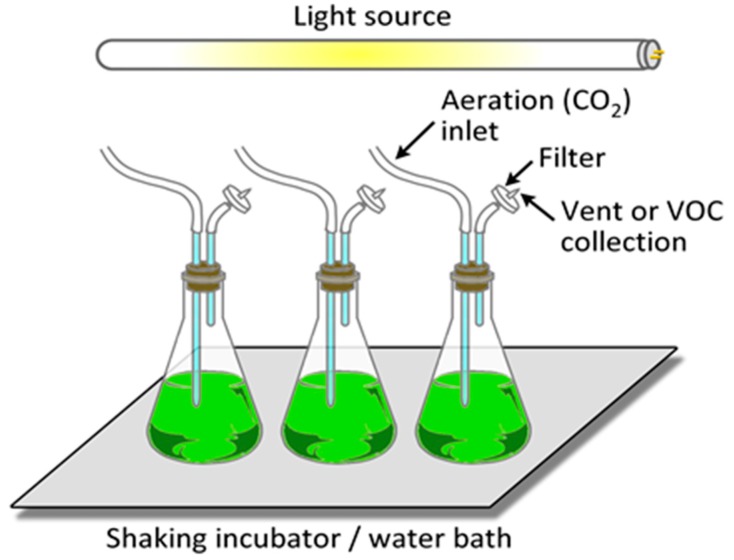
Schematic representation of microalgae culture in the laboratory for collecting VOCs.

For commercial applications, microalgal biology, which in turn determines its productivity, is a key determinant. For a detailed discussion on the various microalgal cultivation methods an excellent guide is recommended [192]. Briefly, strains chosen for commercial cultivation should exhibit fast growth; be predator-, stress-, and disease-resistant; be seasonally tolerant especially in ORPs; produce adequate value-added byproducts; and perform these functions in an environmentally, socially, and economically compliant manner. These are stringent preconditions. Cultivation requirements need to be taken into consideration, which include the availability of C, N, P, trace metals, and vitamins in the nutrients, CO_2_ availability, growth temperature, pH, salinity, light intensity, light availability, and light exposure duration (an ORP concern during cloudy, snowy, stormy, rainy periods), stirring/aeration/turbulence/mixing motion, optimum growth period at which either VOCs or value-added products can be harvested, and so on. A schematic of lab culture of microalgae for harvesting VOCs is shown in Figure 15.

### 6.2. Microalgal VOCs Analysis

Destructive techniques, such as steam distillation as a VOC acquisition method are widely used. Such methods are not considered here since the focus is on VOCs emitted by microalgae in vivo which requires non-invasive and non-destructive technologies such as headspace sampling [193]. It should be noted that microalgal headspace VOC sampling techniques can be adapted from techniques described for plant VOCs collection [194,195,196,197,198]. VOCs from in vivo microalgae may be collected from either microalga cultivated in the lab or from microalgae growing in the marine environment. Once collected, VOCs may be analyzed in the field or transported to an offsite testing lab. Depending upon how VOCs are analyzed, various types of instrumentation are available from lab based equipment to hand-held, field-deployable, gas sensors. Some of these options are discussed below.

### 6.3. VOC Sample Collection

**Figure 16:**
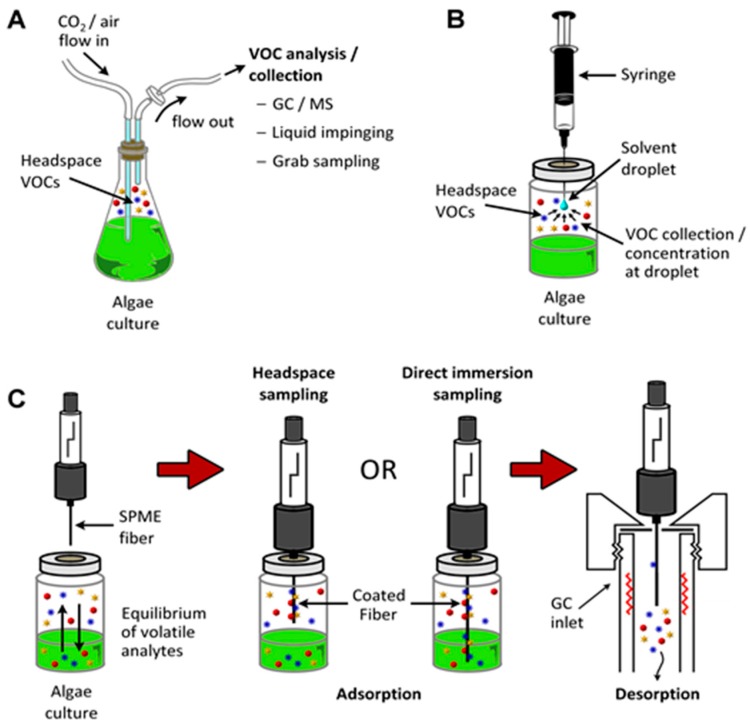
VOC sampling techniques: (**A**) Sampling of headspace by gas flow or collection by liquid impingement or grab sampling; (**B**) SME; (**C**) SPME by adsorption of VOCs from headspace or from both the gas and liquid phase (direct immersion), followed by desorption of VOCs for GC analyses.

For in vivo collection, headspace VOCs have been most widely used [193,199,200]. There are several methods for headspace VOC sampling (Figure 16) such as solvent microextraction (SME), where a drop of solvent is suspended from the tip of a syringe needle above the sample from which VOCs need to be collected. The solvent drop traps and pre-concentrates the VOCs [201]. Non-volatile compounds may be rendered volatile by chemical derivatization (CD) [202]. In solid phase extraction (SPE), VOCs are passed over an adsorbent and then eluted using solvents. An advancement over SPE is SPME [203] which involves the partitioning of analytes between the extraction phase and the sample matrix. The analytes are desorbed from the SPME fiber inside the GC inlet port for analysis [204,205]. SPME is a widely used technique including VOC analyses of marine phytoplankton species [206]. An alternative to SPME is thermal desorption (TD) where the sample is collected onto an adsorbent matrix packed inside a glass or stainless steel tube (TD tube). The tube is heated to release the VOCs into the carrier gas (desorption), from where the volatiles are swept into the GC column for analysis.

Often, it may be necessary to transport the microalgae headspace VOC mixture to an analytical laboratory for GC analysis. In these situations, the gas samples may be collected using SPME fibers, TD tubes, bags made from polymers, or canisters [207]. A variety of factors must be considered in selecting the proper type of container for offsite VOC analysis including sample volume, chemical composition and stability of analytes, storage time and temperature, transportation time, potential contamination or artifacts contributed by the collection container, and the interval between sample collection and analysis. Both SPME and Tenax TA yielded sample recoveries ranging between 95 and 98% following sample storage at room temperature for 24 h [207]. Sample recoveries for bags and canisters were significantly lower, and some bag materials contributed contaminants [207] (due to “outgassing”). If sample volume requirements make collection bags inevitable, then care must be taken to avoid sample loss and/or gratuitous contaminants from the bag material. Nalophan™ and/or PTFE bags may be used to collect VOC emanations from microalgae [208]. Double bagging will minimize VOC losses and minimize ambient confounders [209]. A thicker (~70 μm) wall reduces diffusion losses [209,210]. During storage and shipping, test bags need to be isolated from each other, to avoid cross-contamination due to diffusion from one bag into a neighboring bag. Aseptic techniques are critical during sample collection and handling for reducing bacterial-transformation of microalgal VOCs. Thicker wall bags can store complex VOCs mixtures for up to 24 h at 37 °C and refrigerated samples are stable up to 14 days [211]. Additional stability may be obtained by freezing the bag [212], allowing stability for up to six months with no differences between fresh and frozen VOC samples [213]. Recovery must be monitored using a calibration standard [214].

### 6.4. VOC Analysis

**Figure 17:**
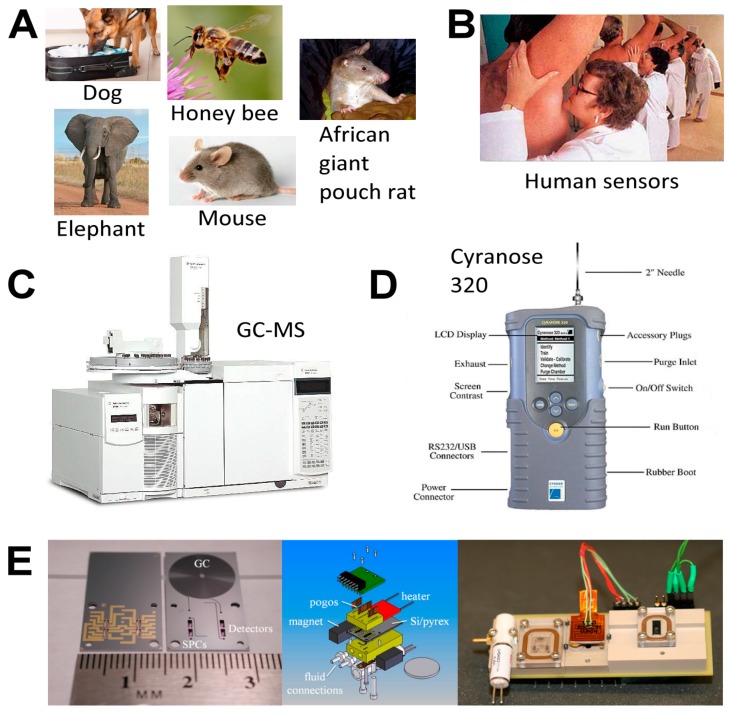
VOC detection. (**A**) Animal sensors; (**B**) Human sensors; (**C**) GC-MS; (**D**) e-Nose; (**E**) μSystems.

Once VOCs have been collected, the next step is the separation of the volatiles and identifying specific molecules in the sample using a variety of techniques (Figure 17).

**Figure 18:**
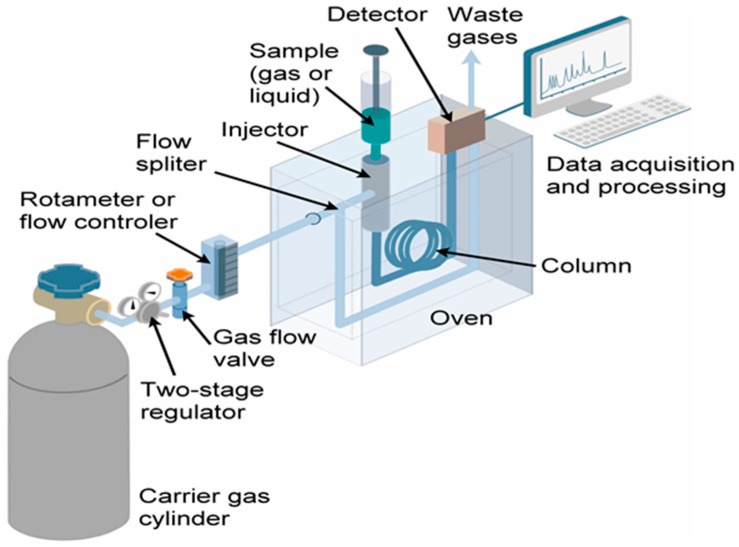
Laboratory scale GC-Detector system is shown schematically.

GC-MS is the most widely used technique for VOC analysis (Figure 18). Briefly, GC involves a mobile phase consisting of an inert carrier gas, such as helium (He), and a stationary phase with varying affinity for the volatiles. The stationary phase is deposited inside a long, narrow tube (GC column). The volatiles interact with the stationary phase as they pass through the column, causing each volatile to emerge (elute) at a different time (retention time) due to different degrees of partitioning of the VOC between the mobile phase and the stationary phase. To separate analytes with a wide range of boiling points, GC column temperature may be increased monotonically (temperature ramping). Depending on the detector used, GC has a linear dynamic range of 4 to 6 orders of magnitude, with sensitivity in parts-per-trillion (ppt). Output signal is converted into a chart (gas chromatogram).

Detectors cited in this review were MS, TCD, FID, FPD, PID, and ECD. In MS, the sample is bombarded by high energy electrons which collide with atoms of the volatile chemical resulting in electrons being ejected, thereby generating a positively charged particle. The charged particles are separated most often magnetically and detected based on their position and relative abundance, yielding a mass spectrum (mass/charge, *m*/*z*). Other detectors employ a variety of physicochemical principles such as thermal conductivity (TCD), flame ionization (FID), photoionization (PID), electron capture (ECD), flame photometric (FPD), pulsed discharge helium ionization (PDHID), and so on. The availability of mass spectral libraries (NIST, for example) enables identification.

A variety of software programs are available for VOC data analysis [215]. Several different data processing techniques are also available such as Principal Component Analysis (PCA). PCA projects the data in two or more dimensions. A plot of PC scores against samples reveals their relationships, identifying principal component(s) with a linear signal. Discriminant Factorial Analysis (DFA) is used for discrimination and identification and may be applied to unknown VOCs. In DFA, variance between different classes is maximized, and the variance within individual classes is minimized. Soft Independent Analysis of Class Analogy (SIMCA) is used to model ‘good/bad’ behavior and is applied as a quality control (QC) against reference (‘good’) compounds. Partial least square analysis (PLSA) and partial least square discriminant analysis (PLSDA) are used for quantitative purposes. Artificial neural network (ANN) back propagation analysis identifies samples accurately [127,216,217,218]. Supplying the biochemical basis via metabolic pathways (vide supra) for the VOCs is an inclusion criterion for microalgae-specific and/or predator-specific volatiles. Pattern recognition and metabolic pathways are facilitated by online databases, repositories of metabolomics/volatilomics, and NIST. In vivo microalgae VOC testing should involve at least three biological replicates with three technical replicates for each biological replicate. Data quality will improve as the sensitivity of VOC analysis increases, since trace VOCs may be detected for improved pattern recognition. Design considerations for assay development were reviewed previously [190].

Conventional GCs are large, heavy, benchtop, expensive, and high power instruments, ill-suited for field VOC monitoring. Onsite, real-time VOC analysis is superior to offsite analysis since sample storage and transportation might limit the sensitivity of reactive or thermally labile VOCs. A systems approach is needed for rapid, specific, sensitive, point-of-use detection of microalgae’s SoH, which may have applications beyond microalgae to other civilian, medical, and military operations. In this low cost, reliable, and portable technology platform, the technical/scientific complexities will be transparent to the end user.

**Figure 19:**
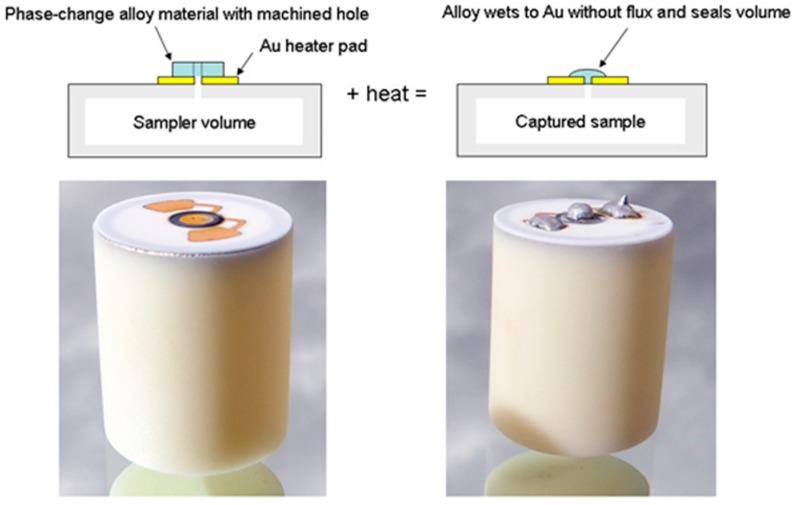
Cross-section and photographs of μSampler and μValve, from [218] with permission.

The first challenge for field VOC analysis is sample collection. Sample acquisition will benefit from low cost, small size/weight/power microsystems. Capturing efficiency will critically impact system performance. The capture-container must be gas-tight to allow transport in case of analysis at a centralized lab. Capture and containment of VOCs is crucial, since VOCs are volatile (Table 1), tending to escape confinement, unless secured hermetically. Commercial SUMMA^®^ canisters are less efficacious when compared to μSamplers [219], the latter yielding a 6000-fold volume reduction. Commercial canisters due to their size and cost, require cleaning prior to re-use. By contrast, mass-microfabricated μSamplers and μValves are inexpensive and may be discarded after a single use, eliminating cross-contamination [219]. Helium leak-tests of μSamplers revealed excellent hermiticity (4.9 × 10^−12^ atm-cc/*s*) meeting and exceeding military specifications (mil-spec) of <1 × 10^−9^ atm-cc/*s* [219]. Phase-change microvalves (Figure 19) employed eutectic alloys which could be actuated at relatively low temperatures of ~72 °C [219].

**Figure 20:**
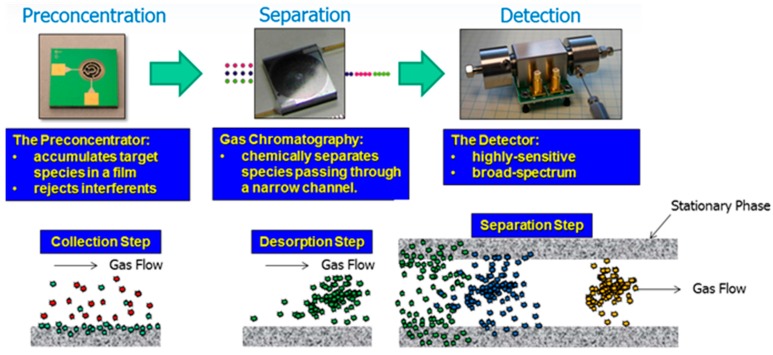
Schematic representation of the operating principles of a portable GC-μSystem.

Terry and Herman [220] first reported in 1979 a micro-GC (μGC) that was fabricated on a silicon (Si) wafer. Since then, several μGC systems have been described for field use with rapid response, small footprint, low-cost, low-power, light weight, battery powered, possessing high resolution with narrow columns having low dead volume and improved sensitivity even with small sample volumes [221,222]. Monolithic integration in Si has enabled system-level miniaturization, low-cost fabrication and assembly, with simpler and smaller fluidic connections [223] (Figure 20). Such systems were developed by Sandia National Laboratories for the detection of chemical warfare agents (CWA). Advancements in μGC systems have been facilitated by similar miniaturization of the VOC detectors [224,225], enabling an integrated microsystem becoming available for AVOC/BVOC analyses [226].

**Figure 21:**
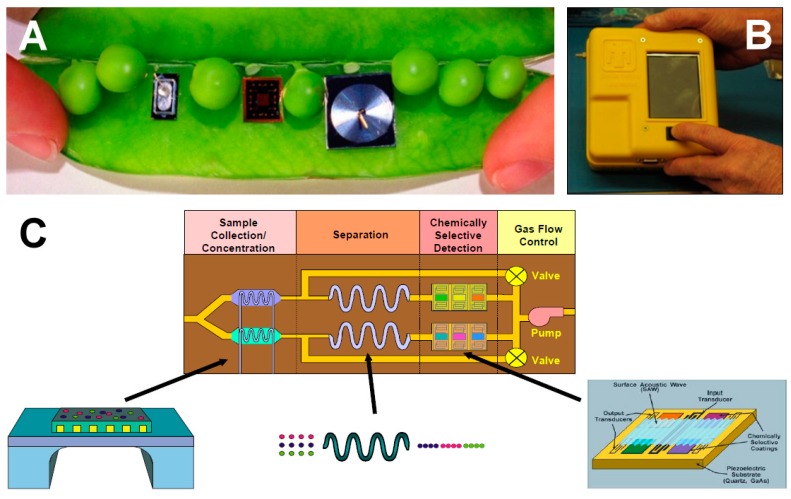
MicroChemLab. (**A**) Principal components of the μGC system fit easily inside a snow-pea pod: (left to right) surface acoustic wave (SAW) detector, μPC, and μGC; (**B**) Packaged MicroChemLab; (**C**) Schematic of the components.

The power of miniaturized, integrated GC μsystems for in vivo microalgae SVOC/VOC analysis is illustrated by Sandia’s MicroChemLab (Figure 21). The MicroChemLab is a handheld chemical analysis system that uses microfabricated components with monolithic integration for combined sample handling (micro-pre-concentrator, μPC), separation (μGC), and detection of VOCs (Figure 21). By tailoring the μPC’s (Figure 21A) chemically-selective film, VOCs are selectively and reversibly adsorbed, while interferents escape. The second stage is the μGC which uses a chemically-selective film to separate the VOC mixture. The column is over 1 m in length, but is formed as a spiral to occupy only 1 cm^2^ of chip area. Heater and temperature probes are affixed to the back of the μGC for controlling the temperature ramp to separate the VOCs. Due to its small footprint and low power requirements, the MicroChemLab has been employed for field detection of VOCs.

As an alternative to surface acoustic wave (SAW) sensor array, an ion mobility spectrometer (IMS) or a Pulsed Discharge Helium Ionization Detector (PDHID) may be substituted [224,225,226,227]. The PDHID uses helium plasma to produce high-energy photons which ionize the VOCs as they elute from the column [228]. This produces free electrons that are collected by a series of electrodes which are in turn connected to conventional charge amplification circuitry. The sensor can detect nearly every compound except Ne, and is capable of detecting sub-parts per billion (sub-ppb) VOC concentrations [228]. Thus, PDHID has almost the sensitivity of MS, but with the size, weight, power usage, and cost factors that allow its field use for in vivo microalgae VOCs analyses. It would also be beneficial to have a suite of other sensors integrated into the VOC microsystem. For example, with in vivo microalgae VOC analyses, it would be helpful to include sensors for non-volatile measurements such as pH, salinity, humidity, light intensity, and perhaps a dedicated CO_2_ sensor for enabling overall systems level investigations to be conducted successfully on a single platform.

The microsystems approach enables several orders of magnitude improvement to sensitivity, with detection limits of parts ppb to ppt using the PDHID [229,230]. In the electron capture mode, PDHID has femtogram (10^−15^) detection limit. These values are orders of magnitude better than e-Nose. Although PDHID sensitivity is not as low as the parts-per-quadrillion (500 ppq; *femtomol*, *fmol*) that can be achieved by MS [231], the combinatorial systems approach of specific VOC identification and pattern recognition is likely to offset the lower detection limit of the PDHID (Table 3).

Rapid field detection of VOCs has also been achieved using electronic nose (e-Nose) [232,233] (Figure 17D). These portable devices consist of an array of conducting polymers, metal oxides, or piezoelectric type sensors that are broadly tuned for detecting various classes of gases. Thus, the e-Nose is a non-specific detector unlike GC-MS. There are a few other portable VOC analysis systems that merit mention. The first of these is the so-called z-Nose [234,235]. This type of analyzer is based on an uncoated quartz-based SAW, and an uncoated piezoelectric quartz crystal that vibrates at a certain frequency. When a volatile molecule alights the sensor, the frequency of SAW changes in a way that is proportional to the concentration of the adsorbing molecule. Since the crystal is in contact with a thermoelectric element, a signal is produced. A library of VOC retention times allows for molecular identification. The z-Nose performs in real-time with ppt-sensitivity and data analysis done is by onboard computational programs [234,235]. The second VOC technology that is portable is the proton transfer reaction-mass spectrometry (PTR-MS). Here, water vapor is ionized in a hollow cathode electrical discharge to produce a stream of pure hydronium ions (H_3_O^+^). The ions enter a drift tube where the ionization of the VOC samples takes place by a protonation reaction as shown below.

H_2_O^+^ + H_2_O → H_3_O^+^ + OH

H_3_O^+^ + VOC → VOCH^+^ + H_2_O

At the end of the drift tube, the protonated VOCs are analyzed for mass using a quadrupole mass analyzer or a time-of-flight (TOF) MS [236]. The PTR-ToF-MS is one of a few analytical techniques that is suitable for online VOCs monitoring. Since PTR-ToF-MS can be carried out without sample preparation [237], it could potentially simplify the process for realtime analysis of VOCs emissions from marine samples [238] and microalgae [239]. Apropos to in vivo marine microalgae VOC analysis in the field, PTR-MS has been used for field measurements of atmospheric VOCs from a variety of air, sea, and ground-based platforms [240]. Finally, Exton et al. [241] demonstrated the use of a dedicated “Fast Isoprene Sensor” (FIS, Hills-Scientific, Boulder, CO, USA) for quantitative analysis of isoprene from the microalgae *Thalassiosira weisflogii* and *Emiliania huxleyi*. The FIS operates on formaldehyde (HCHO) and glyoxal (HCOCHO) generation following a reaction between isoprene and O_3_. Both products are in the excited state. Upon relaxation, they emit light at ~490 nm and ~550 nm, respectively, detected by a chemiluminescence detector. The limit of detection (LOD) was 0.02 nM (0.5 ppbv) isoprene with production rates that were detectable even at a low 0.59 nmol/h from these two microalgae [241]. Interestingly, the reaction product formaldehyde (between isoprene and ozone) can also be detected using PDHID with nM sensitivity [242,243,244], and is thus suitable for field analysis, unlike the FIS, which due to size/power considerations, requires lab operations [241].

## 7. Future Perspectives

**Figure 22:**
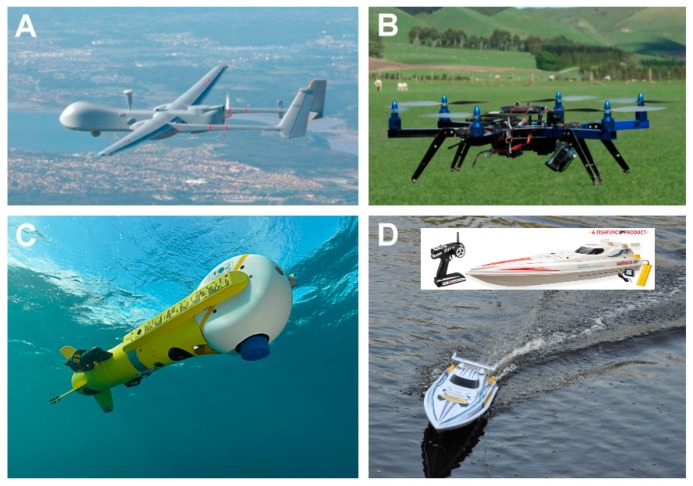
UAV, AUV, and ROV. (**A**) UAV (http://www.evolvsys.cz/news/news_2006.html); (**B**) drone to measure crop properties (http://www.dronemagazine.it); (**C**) AUV to detect and dispose underwater mines (http://www.ecagroup.com); (**D**) hobby boat that could be used to carry instrumentation to measure VOCs (Radio Ranger Fishing Boat, http://www.rcfishingworld.com/).

If successful, such portable test beds for marine in vivo microalgae VOCs could be amenable to manned or unmanned, remotely controlled operations. Satellite technology has supplied ocean maps of phytoplankton distributions [245] and BVOC emissions over vast areas [246]. However, satellite sensors can be costly and unsuited for routine use. Small unmanned aerial vehicles (UAV) have been used for taking air quality and aerosol distribution measurements [246,247] (Figure 22A,B). Due to their small size and low cost, UAVs or drones will be restricted in terms of size, weight, power, and portability (SWAPP) factors relating to payload, flight speed, and endurance (flight time). Consequently, lightweight, small, low power VOC sensors such as the microsystems discussed above will be critical for placing aboard UAVs. Although the potential of UAVs for VOC monitoring has passed the technological feasibility test [246,247], the real challenge might well be country-specific policies and regulations governing the deployment of UAVs [247].

There are other options for VOCs monitoring in an underwater marine environment (Figure 22C,D). These include remote operated vehicles (ROV), human occupied vehicles (HOV), autonomous underwater vehicles (AUV), manned submersibles, submarines, human divers, tethered buoys, towfish, and so on [248]. The availability of MS that can take measurements underwater (membrane inlet mass spectrometer, MIMS) has made underwater VOC analysis routine [248]. AUVs have been used to measure phytoplankton VOC emissions for monitoring marine pollution and surveilling the emergence of harmful algal blooms (HAB) [249,250,251]. There is a priori no reason to not be able to mount microsystems (vide supra) aboard AUVs/UAVs to expand autonomous investigations into in vivo microalgal VOC emanations. It is hoped that this review will stimulate further research and development (R&D) activities in the exciting VOC frontiers in vivo microalgae, and beyond.

## 8. Conclusions

Herein the reports of VOC emissions from in vivo (living, growing, and naturally dying) microalgae were reviewed. Much of the literature addressing microalgae VOC production focuses on the implication of these biogenic emissions towards atmospheric and marine geochemical content (e.g., biogenic sulfur, halocarbons, etc.). Due to the significant prevalence of microalgae, these emissions may influence the global environment and ecology. Where known, biosynthetic/metabolic pathways associated with the production and emission of microalgae VOCs were presented [100,101,106,107,108,123]. These pathways are susceptible to abiotic (for example, light stress [252] and ionic strength [253]), and biotic influences (for example, growth phase [254]). Such pathways provide context regarding the biological and biochemical roles of VOCs in the microorganism. Thus, understanding VOC profiles and the volatilome/volatome of microalgae in vivo may provide a non-invasive means to evaluate their physiology, pathology, and ecology in real-time and predict/prevent algal pond crashes. To date, only a few direct studies of the physiological significance of VOC emissions from microalgae have been published. These studies do show VOC-based intra-species communication between microalgae, and inter-species communication between microalgae and bacteria [255,256], for example by the release of aldehydes [257] due to wounding [258]. Still, many questions regarding the purpose and impact of emitted VOCs on microalgae physiology remain unanswered.

Major challenges remain for studying in vivo microalgae VOCs. Controlling the many factors that contribute to changes in metabolic behaviors that influence VOC production and emission is challenging even under laboratory conditions where small cultures can be grown under carefully defined and known/measurable parameters. This becomes increasingly difficult during commercial, large scale production, and nearly impossible to manage in the natural marine habitat. The use of axenic cultures and strains is important to correctly attribute measured VOCs to a specific alga, and not a pollutant or contaminate species. Despite these challenges, advancements in culture techniques and careful design of experiments, including statistically meaningful replicates and the use of pattern-analysis techniques, can provide critical information that will contribute towards the understanding of the microalgae volatilome.

Finally, this review offered various tools and techniques for the collection, storage, transport, and analysis of VOCs from microalgae. Recent advances have been reported in portable technologies for in field sample collection and storage, allowing gold standard lab analysis of VOCs captured far outside the laboratory. Also, exciting advances in microsystems for in vivo VOC identification and analysis outside the laboratory have enabled the real-time detection of trace levels of VOCs using hand-held devices. These advances will accelerate efforts into identifying and understanding the in vivo microalgae volatilome and open other opportunities that go beyond point-of-use detection of microalgae’s SoH for a variety of societal benefits and commercial opportunities.

## Figures and Tables

**Table 1:** General properties of VOCs.

Class	Abbreviation	Vapor Pressure (VP) (*p*) and Atmospheric Pressure (*P_atm_*)	VP (mm Hg) at 25 °C	Boiling Point (°C)	Example
Very Volatile Organic Compound	VVOC	*p* >> *P_atm_*	>380	<0 up to 50	Propane
Volatile Organic Compound	VOC	*p* > *P_atm_*	0.1 to 380	~50 to 250	Acetone
Semi-Volatile Organic Compound	SVOC	*p* = *P_atm_*	10^−7^ to 10^−1^	250 to 400	Plasticizers
Non-Volatile Organic Compound	NVOC	*p* << *P_atm_*	<10^−7^	Not applicable	Glucose C_6_H_12_O_6_

**Table 2:** Biochemistry of certain VOCs.

VOC	Metabolic Basis
H_2_, CH_4_ (Hydrogen, Methane)	Carbohydrate
C_2_H_6_CO (Acetone)	Acetoacetate decarboxylation
NH_3_, CH_3_NH_2_ (Ammonia, Methylamine)	Protein
H/Cs, C_5_H_12_, C_2_H_6_, C_2_H_4_ (Hydrocarbons)	Lipid peroxidation
NO (Nitric oxide)	Nitric oxide synthase
C_2_H_5_OH, CS_2_, COS (Carbonyl sulfide)	Intestinal bacteria
CO (Carbon monoxide)	Heme catabolism
CH_3_SH, C_2_H_6_S (Methane/Ethane thiols)	Methionine metabolism
CH_3_CHO (Acetaldehyde)	Ethanol metabolism
C_5_H_10_ (Cyclopentane)	Cholesterol metabolism

**Table 3:** Comparison of portable e-Nose and microsystems with GC-MS.

Property	Electronic Nose	μGC/μPC/μPDHID	GC-MS
Specific VOC identified?	No	Yes	Yes
Instruments comparable?	No	Yes	No
Signals comparable?	No	Yes	No
Onboard pattern analysis	Yes	Yes	No
Sensitivity limits	ppm-to-ppb	ppb-to-ppt	ppt-ppq
Sensor drifts?	Yes	No	No
Sensor poisoning?	Yes	No	No
Responses are variable?	Yes	No	No
Individual VOC quantifiable?	No	Yes	Yes
Power requirements	Low-to-moderate	Low-to-moderate	High
Skill/training of operator	Moderate	Moderate	High
Portability?	Yes	Yes	No
